# Workplace indicators and well-being among oral health professionals in HRSA-funded health centres

**DOI:** 10.1093/haschl/qxag211

**Published:** 2026-08-13

**Authors:** Jinman Pang, Simona Surdu, Sage Shirey, Jean Moore

**Affiliations:** Oral Health Workforce Research Center, Center for Health Workforce Studies, College of Integrated Health Sciences, University at Albany, State University of New York (SUNY), Albany, NY 12222, USA; Oral Health Workforce Research Center, Center for Health Workforce Studies, College of Integrated Health Sciences, University at Albany, State University of New York (SUNY), Albany, NY 12222, USA; Oral Health Workforce Research Center, Center for Health Workforce Studies, College of Integrated Health Sciences, University at Albany, State University of New York (SUNY), Albany, NY 12222, USA; Oral Health Workforce Research Center, Center for Health Workforce Studies, College of Integrated Health Sciences, University at Albany, State University of New York (SUNY), Albany, NY 12222, USA

**Keywords:** dental assistants, dental hygienists, dentists, job satisfaction, engagement, burnout, retention, workplace drivers, workload, moral distress, safety net

## Abstract

**Introduction:**

Oral health provider well-being is critical to sustaining the HRSA Health Centre (HRSA-HC) workforce, yet limited evidence exists on the drivers of well-being outcomes specifically among dental assistants (DAs), dental hygienists (DHs), and dentists in safety-net settings.

**Methods:**

This cross-sectional study used data from the 2022 HRSA Health Centre Workforce Well-being Survey among 2671 oral health providers (DAs = 1385; DHs = 564; dentists = 722). Well-being outcomes for job satisfaction, engagement, and burnout were calculated as mean Likert scores and categorized into tertiles; intention to stay was dichotomized. Descriptive statistics, multinomial logistic regression, and multivariable logistic regression with backward stepwise selection were used to examine associations between 16 well-being drivers and four outcomes separately by provider type.

**Results:**

DHs reported the most favourable well-being outcomes, with the highest levels of job satisfaction, engagement, and intention to stay, while burnout was similar across provider types. Work-life balance, meaningfulness, and professional growth were consistently associated with more favourable well-being outcomes and lower burnout, while workload and moral distress were related to higher burnout and poorer well-being across most provider types. Additional provider-specific drivers were also identified.

**Conclusion:**

These findings underscore the need for both universal and provider-specific organizational strategies to strengthen oral health workforce well-being and retention at HRSA-HCs.

## Introduction

Health Resources and Services Administration-supported health centres (HRSA-HCs) are a critical resource for individuals who may otherwise lack access to care. In 2024, HRSA-HCs provided services for 32.4 million patients across the United States (US), roughly two-thirds of whom were living at or below the federal poverty level.^1,2^ Unfortunately, only 20.9% of these patients received oral health services compared with 84.9% who received medical care services.^1^ Dental provider staffing in HRSA-HCs is a major contributor to this disparity. The 2025 workforce projections from HRSA's National Centre for Health Workforce estimated shortages of dentists and dental hygienists (DHs) by 2038.^3^ In addition, most participants responding to the American Dental Association's 4th Quarter 2025 Economic Outlook survey reported that dental assistants (DAs) (70.4%) and DHs (88.3%) were either “very” or “extremely” challenging to recruit.^4^ While these findings referred to a wide variety of oral health settings, they highlight the challenges with recruiting providers and emphasize the importance of retaining current staff.

Burnout continues to affect oral health care workers and is associated with retention challenges at HRSA-HCs.^5^ One study found that 13% of participating dentists across settings experienced high burnout,^6^ while another found that 58% reported experiencing burnout a few times per week or more.^7^ Similarly, nearly half of participating DAs reported feeling overwhelmed by their current workload.^8^ Another study of DHs found that nearly a third of the participants were at high risk of burnout,^9^ and research suggests that burnout is associated with an increased intention to leave among DHs.^10-12^ Additional drivers of burnout and intention to leave among oral health professionals include the physical demands of the job (such as bending over and performing fine motor skills for extended periods of time), poor workplace culture, fewer benefits, and workload.^8,10,11,13-15^ Studies also suggest that factors such as poor workplace culture, inadequate compensation, moral distress, and insufficient staffing are associated with higher burnout and greater intention to leave among nursing assistants (NAs), registered nurses (RNs), and physicians.^16-19^ Addressing these drivers is helpful for improving oral health provider staffing at HRSA-HCs.

Conversely, increasing job satisfaction and work engagement may help improve retention in HRSA-HCs. One study found that work engagement was negatively associated with burnout among dentists,^6^ while other research suggested that higher job satisfaction is negatively associated with both burnout and intention to leave among DHs.^12,15^ Higher job engagement was also found to be associated with lower stress among DHs.^15^ One study found that 80.3% of DHs and 81.2% of DAs were either “very satisfied” or “somewhat satisfied” with their job.^20^ Drivers of job satisfaction for oral health providers may also apply to those working in HRSA-HCs. Studies suggest that a positive workplace culture, opportunities to help patients, and professional growth have been associated with higher job satisfaction, while overwork and insufficient pay have been associated with lower satisfaction, particularly for DAs, who tend to be paid less than other oral health professionals.^10,15,20,21^

Addressing drivers of well-being outcomes, including job satisfaction, burnout, engagement, and intention to stay, may inform efforts to strengthen oral health service capacity in HRSA-HCs. Unfortunately, prior studies have typically focused on limited well-being outcomes, examined specific oral health professionals, and relied on national-level data that may not capture the unique experiences of providers working in HRSA-HCs.^13,20^ Additionally, although the 2022 HRSA Health Centre Workforce Well-being Survey examined factors associated with well-being outcomes among providers overall, it did not differentiate by provider type.^5^ As a result, it remains unclear whether these workforce factors are similarly associated with well-being among oral health providers.

The Job Demands-Resources (JD-R) framework provides a conceptual basis for examining workplace factors associated with workers well-being.^22,23^ Under this framework, job demands require sustained physical or psychological effort and may be associated with strain, whereas job resources relate to motivation, engagement, and continued employment. In this study, workload and moral distress were conceptualized as job demands, while factors like teamwork, supervision, recognition, adequate resources, and meaningfulness of work, professional growth, and work-life balance were conceptualized as job resources. Because DAs, DHs, and dentists differ in their occupational responsibilities, autonomy, compensation, and advancement opportunities, these associations between these workplace factors and well-being outcomes may vary across provider types. Guided by this framework, the purpose of this study was to analyse these data to examine factors related to well-being outcomes among DAs, DHs, and dentists and to assess how these associations vary by provider and HRSA-HC characteristics.

## Data and methods

### Data

This cross-sectional study used publicly available data from the 2022 HRSA Health Centre Workforce Well-being Survey (HRSA Data Downloads: https://data.hrsa.gov/data/download?data=SCH#). The survey was administered to all full- and part-time staff employed at HRSA-HCs between November 2022 and February 2023.^5^ Of the 1481 HRSA-HCs nationwide, 694 (46.9%) participated in this survey. Participating centres compared with not participating were more likely to be 330-funded health centres, medium in size, and serve higher proportions of patients covered by Medicaid. Over 143 000 survey emails were distributed, and 52 568 staff members participated and completed this survey, yielding an overall response rate of 37%. Respondents included 1435 DAs, 596 DHs, and 807 dentists; profession-specific response rates were not available. Overall response rates were significantly higher among rural health centres, smaller centres, and Look-Alike health centres (organizations that meet HRSA's Health Centre Program requirements but do not receive Section 330 grant funding).^24^ Additional details on survey design, data collection procedures, questionnaire content, provider demographics, health centre characteristics, and occupational factors are provided in the technical report.^5^ The Institutional Review Board determined that this study did not meet the regulatory definition of human subjects research (ie, it involved the secondary analysis of de-identified data).

### Measurement of outcome variables

This study examined four workforce well-being outcomes: job satisfaction, burnout, engagement, and intention to stay at HRSA-HCs. Job satisfaction was measured using five survey questions, burnout using 16 questions, engagement using six questions, and intention to stay using one question. All survey questions were assessed on a 6-point Likert scale. Higher scores indicated stronger agreement for job satisfaction, engagement, and intention to stay; while for burnout items, higher scores indicated stronger disagreement. For analytic consistency, and by following the 2022 HRSA Health Centre Workforce Well-being Survey, burnout items were reverse coded prior to analysis so that higher scores reflected stronger agreement across all outcomes.

Each outcome was calculated as a mean Likert score based on its corresponding survey questions. To address missing data (ie, respondents who answered some but not all survey questions for a specific outcome), we established a minimum response threshold for inclusion in the mean score calculation. This threshold was determined based on the maximum number of unanswered survey questions observed among all oral health providers for each outcome. Respondents were included in the analysis if they were missing fewer than half of this threshold. Based on the distribution of mean Likert scores, job satisfaction, engagement, and burnout were categorized into tertiles to distinguish respondents with relatively low, intermediate, and high levels of each outcome without assuming a linear association across the full score range. Intention to stay was dichotomized based on Likert scores as low (1-4) or high (5-6), distinguishing weaker or uncertain intention to remain from stronger intention to stay.

### Measurement of well-being drivers

Survey respondents were asked about 16 well-being drivers, including work team (communication, collaboration, and cohesion; 8 survey questions), supervision (support from immediate supervisors; 5 questions), leadership (support from senior leaders; 3 questions), positive workplace culture (well-being, inclusion, nondiscrimination; 12 questions), social support (workplace help; 4 questions), recognition (workplace appreciation; 5 questions), supportive health centre process (administration, workflows, quality of care; 5 questions), training provided (job training supported by the health centre; 3 questions), adequate resources (staffing, supplies, infrastructure; 7 questions), mission orientation (alignment of organization and individual goals; 4 questions), meaningfulness (purpose and engagement; 5 questions), compensation and benefits (satisfaction with pay and benefits; 4 questions), professional growth (professional development and promotion; 4 questions), workload (work demands and level of control; 6 questions), work-life balance (balance of work and personal time; 5 questions), and moral distress (conflict between work demands and personal values; 4 questions). Consistent with the JD-R framework, workload and moral distress were conceptualized as job demands, whereas the remaining workplace indicators were conceptualized as job resources. Each driver was measured using at least three survey questions assessed on the same 6-point Likert scale. For most drivers, higher scores indicated stronger agreement. Workload and moral distress questions were originally coded so that higher scores indicated stronger disagreement and lower levels of these job demands. These items were therefore reverse coded so that higher scores represented greater workload and moral distress. Consequently, higher scores for all workplace indicators represented greater levels of the construct named. Mean Likert scores were calculated for each driver using the same missing data approach described above. Scores were subsequently categorized into tertiles or dichotomized at the median, with dichotomization applied to variables with limited variability (eg, mission orientation and meaningfulness).

### Measurement of covariates

Potential confounding variables were obtained from the original data categories, including survey respondent age (under 40 years and 40 years or older), gender (male and female or other), race and ethnicity (White non-Hispanic and all other racial and ethnic groups), marital status (married, never married, and previously married or separated), urban or rural location of the health centre, health centre size (small and large), health centre funding (CHC-only or Look-Alike and multiply funded), and HRSA region. Highest education was reclassified separately by provider type according to standard education levels: for DAs (high school or less; certification or some college; associate, bachelors, or postgraduate degree), for DHs (associate or bachelor's degree; postgraduate degree), and for dentists (postgraduate degree only).

### Statistical analysis

Survey participants with complete data were included in the final analytic samples: 1385 DAs, 564 DHs, and 722 dentists (missing values for all variables were < 2%). Descriptive statistics, ANOVA, and Pearson's chi-square tests evaluated differences in oral health professionals well-being outcomes and drivers, demographics, and health centre characteristics by provider types. Multinomial logistic regression models assessed associations between three-level outcomes and well-being drivers, demographics, and health centre characteristics for each provider group separately, with results reported as relative risk ratios (RRRs) and 95% confidence intervals (CIs). Binary logistic regression assessed intention to stay, with results reported as odds ratios (ORs) and 95% CIs. Because the models considered 16 well-being drivers and were estimated separately for each provider type and well-being outcome, all workplace factors were initially included, and backward stepwise selection was used to obtain parsimonious models. Drivers with *P* < .10 were retained. Age, race and ethnicity, highest education, and marital status were prespecified as potential confounders and retained in all models regardless of statistical significance. All analyses were conducted using STATA version 19.0 (Stata Corp., College Station, TX), with statistical significance set at 0.05.

## Results

Table 1 presents the mean scores for the four well-being outcomes and 16 drivers by oral health provider type at HRSA-HCs, with higher scores indicating more favourable outcomes except for burnout. Overall, DHs reported significantly higher levels of job satisfaction (4.80), engagement (4.98), and intention to stay (5.19) compared with DAs and dentists, who had slightly lower scores across these measures. Burnout levels did not differ significantly by oral health provider type (3.03-3.10). Cronbach's alpha coefficients exceeded 0.80 for each well-being outcome and provider type, indicating high internal consistency among the survey questions. Among all oral health providers, the highest-scoring job resources were consistently work meaningfulness, mission orientation, and social support, while compensation and benefits was the lowest-scoring job resource. Among job demands, workload and moral distress had relatively low mean scores (Table 1). Dental hygienists reported the highest mean scores overall (eg, meaningfulness 5.53, mission orientation 5.46), with values significantly higher than those of dentists and DAs. Most other drivers scored between 4.00 and 5.00 across groups. Cronbach's alpha coefficients indicated generally acceptable internal consistency for all well-being drivers (>0.65), except for social support among DAs (*α* = .596).

**Table 1.** Characteristics of survey respondents in HRSA-HCs by oral health provider type, 2022-2023.

	Dental assistants (*n* = 1385)		Dental hygienists (*n* = 564)		Dentists (*n* = 722)		*P*-value
Well-being outcomes (no. of items)	No.	%; Mean^e^ (alpha)	No.	%; Mean (alpha)	No.	%; Mean (alpha)	
Job Satisfaction (5)	1385	4.57 (0.897)	564	4.80 (0.905)	722	4.64 (0.905)	.**0002**
Burnout (16)	1385	3.10 (0.933)	564	3.03 (0.938)	722	3.09 (0.935)	.2772
Engagement (6)	1385	4.88 (0.842)	563	4.98 (0.847)	721	4.79 (0.877)	.**0025**
Intention to Stay (1)	1383	4.87 (NA)	564	5.19 (NA)	721	5.02 (NA)	**<**.**001**
**Well-being drivers (no. of items)**							
Work Team (8)	1385	4.66 (0.922)	564	4.78 (0.922)	722	4.94 (0.918)	**<**.**001**
Supervision (5)	1385	4.82 (0.896)	564	4.96 (0.870)	722	4.92 (0.903)	.**0269**
Leadership (3)	1383	4.36 (0.760)	564	4.36 (0.756)	722	4.27 (0.775)	.3037
Positive Workplace Culture (12)	1385	4.73 (0.926)	564	4.86 (0.912)	722	4.93 (0.933)	**<**.**001**
Social Support (4)	1385	4.91 (0.586)	564	5.00 (0.687)	721	5.08 (0.690)	.**0001**
Recognition (5)	1385	4.63 (0.742)	564	4.70 (0.752)	722	4.74 (0.752)	.**0225**
Supportive Health Centre Processes (5)	1171	4.30 (0.702)	523	4.07 (0.752)	709	4.04 (0.719)	**<**.**001**
Training Provided (3)	1385	4.36 (0.734)	563	4.64 (0.677)	722	4.56 (0.717)	**<**.**001**
Adequate Resources (7)	1385	4.71 (0.905)	564	4.73 (0.912)	722	4.64 (0.922)	.117
Mission Orientation (4)	1384	5.25 (0.809)	564	5.46 (0.779)	722	5.45 (0.799)	**<**.**001**
Meaningfulness (5)	1385	5.31 (0.862)	564	5.53 (0.848)	721	5.42 (0.873)	**<**.**001**
Compensation & Benefits (4)	1384	3.61 (0.755)	564	3.87 (0.724)	722	3.82 (0.728)	**<**.**001**
Professional Growth (4)	1384	4.55 (0.793)	562	4.67 (0.791)	722	4.59 (0.808)	.0787
Workload (6)	1384	2.90 (0.714)	564	2.89 (0.712)	722	2.93 (0.745)	.6769
Work-life Balance (5)	1385	4.26 (0.724)	564	4.53 (0.776)	722	4.28 (0.775)	**<**.**001**
Moral Distress (4)	1385	2.67 (0.731)	564	2.66 (0.748)	722	2.79 (0.749)	.**0168**
**Control Variables**							
Age	**1380**		**564**		**722**		**<**.**001**
Under 40	785	56.9%	231	41.0%	321	44.5%	
40 and older	595	43.1%	333	59.0%	401	55.5%	
Gender	**1385**		**564**		**722**		**<**.**001**
Male	74	5.3%	25	4.4%	270	37.4%	
Female/Other^a^	1311	94.7%	539	95.6%	452	62.6%	
Race/Ethnicity	**1383**		**560**		**716**		**<**.**001**
White Non-Hispanic	548	39.6%	396	70.7%	363	50.7%	
All Other Race Ethnicities^b^	835	60.4%	164	29.3%	353	49.3%	
Highest Education	**1385**		**564**		**722**		**<**.**001**
High school or less	207	14.9%	0	0.0%	0	0.0%	
Certification or Some College	792	57.2%	0	0.0%	0	0.0%	
Associate or Bachelor's Degree or Post Graduate Degree^c^	386	27.9%	0	0.0%	0	0.0%	
Associate or Bachelor's Degree	0	0.0%	537	95.2%	0	0.0%	
Post Graduate Degree	0	0.0%	27	4.8%	722	100.0%	
Marital Status	**1303**		**539**		**664**		**<**.**001**
Married	427	32.8%	66	12.2%	113	17.0%	
Never Married	696	53.4%	387	71.8%	512	77.1%	
Previously Married/Separated	180	13.8%	86	16.0%	39	5.9%	
Urban/Rural	**1385**		**564**		**722**		**<**.**001**
Rural	562	40.6%	263	46.6%	211	29.2%	
Urban	823	59.4%	301	53.4%	511	70.8%	
Health Centre Size	**1385**		**564**		**722**		**<**.**001**
Small	265	19.1%	122	21.6%	90	12.5%	
Large	1120	80.9%	442	78.4%	632	87.5%	
Health Centre Funding	**1435**		**564**		**722**		.**004**
CHC-only or Look-Alike^d^	769	55.5%	358	63.5%	404	56.0%	
Multiply funded	616	44.5%	206	36.5%	318	44.0%	
HRSA Region	**1385**		**564**		**722**		**<**.**001**
Region 1 (CT, ME, MA, NH, RI, VT)	83	6.0%	47	8.3%	83	11.5%	
Region 2 (NJ, NY, PR, Virgin Islands)	110	7.9%	56	9.9%	74	10.2%	
Region 3 (DE, DC, MD, PA, VA, WV)	109	7.9%	61	10.8%	50	6.9%	
Region 4 (AL, FL, GA, KY, MS, NC, SC, TN)	100	7.2%	51	9.0%	66	9.1%	
Region 5 (IL, IN, MI, MN, OH, WI)	220	15.9%	117	20.7%	89	12.3%	
Region 6 (AR, LA, NM, OK, TX)	108	7.8%	41	7.3%	51	7.1%	
Region 7 (IA, MO, NE, KS)	121	8.7%	64	11.3%	50	6.9%	
Region 8 (CO, MT, ND, SD, UT, WY)	47	3.4%	21	3.7%	29	4.0%	
Region 9 (AZ, CA, HI, NV, American Samoa, Guam, Northern Mariana Islands, Federated States of Micronesia, Marshall Islands, Palau)	378	27.3%	56	9.9%	162	22.4%	
Region 10 (AK, ID, OR, WA)	109	7.9%	50	8.9%	68	9.4%	

Analysis of variance (ANOVA) was used to assess differences in continuous well-being outcomes and drivers across oral health provider types (DAs, DHs, and dentists), while Pearson chi-square tests were used to evaluate differences in categorical individual demographic and health centre characteristics across these provider groups; *P* < .05 was considered significant. Bold values indicate the total number of observations for each variable within each provider, along with the significance level for differences.

Source: Health Resources and Services Administration (HRSA) Health Centre Workforce Well-being Survey, 2022-2023.

^a^Female/Other, female, female-to-male (FTM)/transgender male/trans man, male-to-female (MTF)/transgender female/trans women, genderqueer, neither exclusively male or female, something else, and don’t know/not sure based on gender identity.

^b^ll Other Race Ethnicities, Hispanic, Black Non-Hispanic, and Other Non-Hispanic.

^c^Associate or Bachelor's Degree or Post Graduate Degree, Associate's degree (eg, AA, AS), Bachelor's degree (eg, BA, BS), Master's degree (eg, MA, MS, MBA), Doctoral/Professional degree (eg, MD/DO, DMD/DDS, PhD).

^d^CHC-only or Look-Alike, Community Health Centre (CHC)-Only.

^e^Survey questions were assessed on a 6-point Likert scale ranging from (1) strongly disagree to (6) strongly agree. Items were reverse scored as needed so that higher scores represented greater levels of the construct named. Thus, higher burnout, workload, and moral distress scores indicate less favourable well-being outcomes or workplace conditions, whereas higher scores for the remaining outcomes and workplace factors indicate more favourable outcomes or conditions.

At HRSA-HCs, DAs were significantly younger (56.9% under 40), and more likely to be from racial or ethnic minority groups (60.4%) compared with other provider types. In contrast, DHs and dentists were generally older (59.0% and 55.5% ≥ 40, respectively), and were predominantly white (70.7% and 50.7%, respectively) (Table 1). Most DA, DHs, and dentists were female (94.7%, 95.6%, and 62.6%, respectively) and worked in urban settings (59.4%, 53.4%, and 70.8%, respectively).

**Figure 1.**
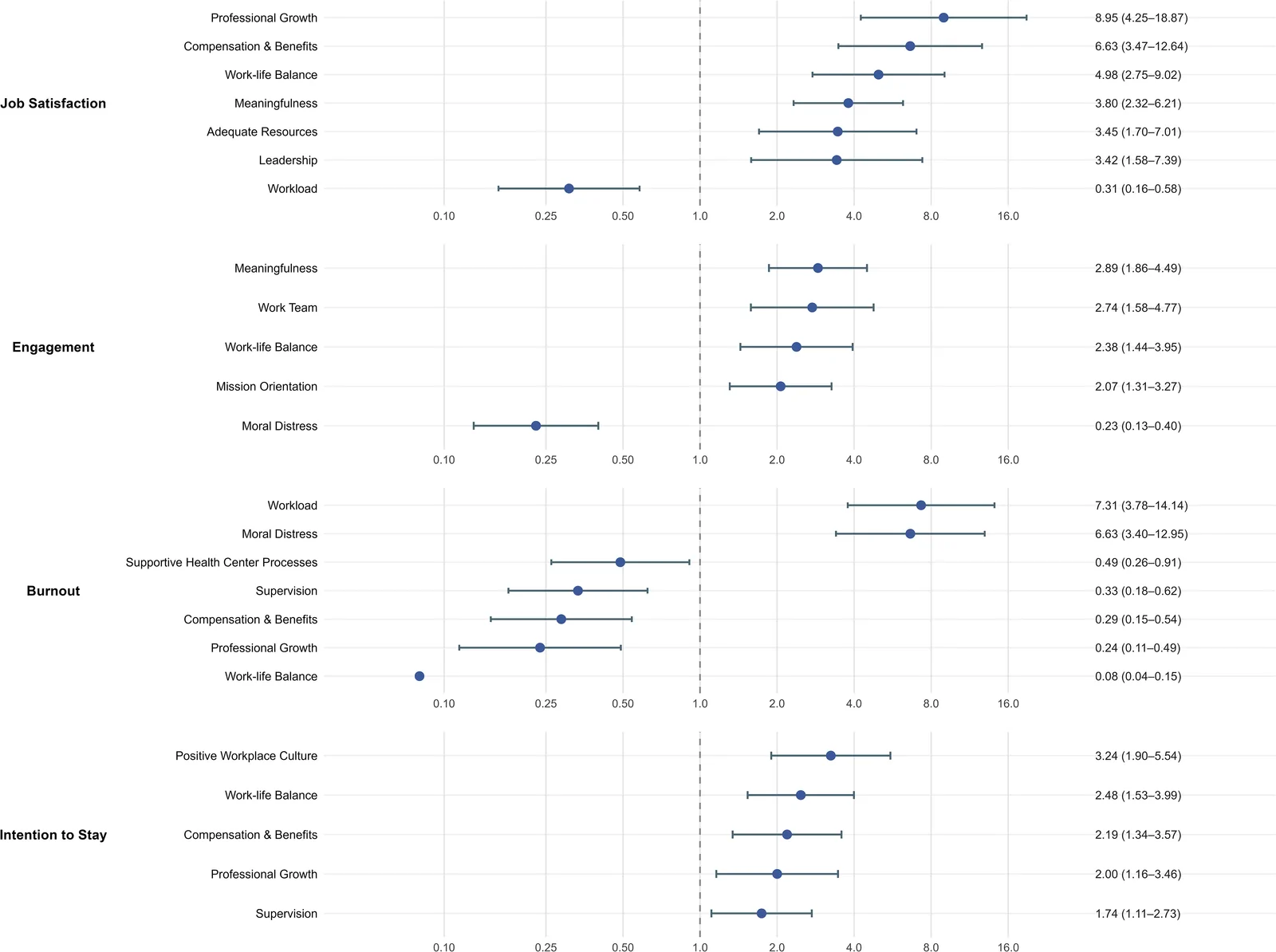
Significant adjusted associations of workforce well-being drivers with job satisfaction, engagement, intention to stay, and burnout Among dental assistants in HRSA-HCs, 2022-2023. Source: Health Resources and Services Administration (HRSA) Health Centre Workforce Well-being Survey, 2022-2023. Note: Complete model results are presented in Table S1. Multinomial logistic regression models (relative risk ratios [RRRs] and 95% confidence intervals [CIs]) estimated the associations between three-category outcomes and well-being drivers, adjusted for demographic and health centre characteristics for dental assistants. Binary logistic regression (odds ratios [ORs] and 95% confidence intervals [CIs]) assessed intention to stay. Models were estimated using backward stepwise selection, with variables entered at a significance level of 0.10; age, race and ethnicity, highest education attainment, and marital status were retained in all models. This figure presents adjusted estimates comparing the highest vs lowest level of each well-being driver or outcome, defined as the third vs first tertile or higher vs lower dichotomized level, and is restricted to statistically significant associations (*P* < .05). Higher scores correspond to higher levels of the indicated construct. As workload, moral distress, and burnout are unfavourable constructs, estimates >1 in the burnout panel reflect greater burnout (less favourable outcomes), whereas estimates >1 in the job satisfaction, engagement, and intention to stay panels indicate more favourable outcomes. The reference line at 1 indicates no association.

**Figure 2.**
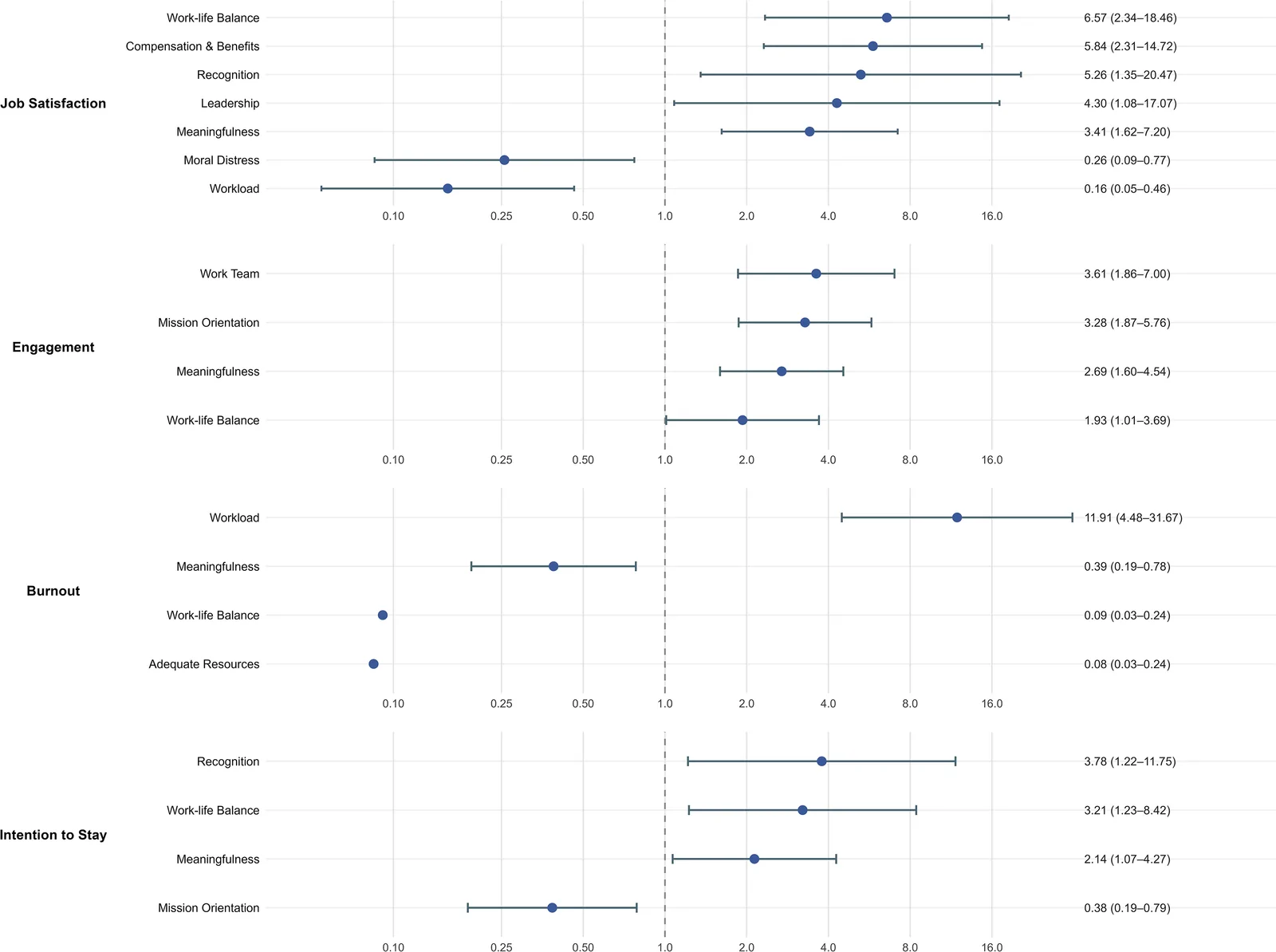
Significant adjusted associations of workforce well-being drivers with job satisfaction, engagement, intention to stay, and burnout Among dental hygienists in HRSA-HCs, 2022-2023. Source: Health Resources and Services Administration (HRSA) Health Centre Workforce Well-being Survey, 2022-2023. Note: Complete model results are presented in Table S2. Multinomial logistic regression models (relative risk ratios [RRRs] and 95% confidence intervals [CIs]) estimated the associations between three-category outcomes and well-being drivers, adjusted for demographic and health centre characteristics for dental hygienists. Binary logistic regression (odds ratios [ORs] and 95% confidence intervals [CIs]) assessed intention to stay. Models were estimated using backward stepwise selection, with variables entered at a significance level of 0.10; age, race and ethnicity, highest education attainment, and marital status were retained in all models. This figure presents adjusted estimates comparing the highest vs lowest level of each well-being driver or outcome, defined as the third vs first tertile or higher vs lower dichotomized level, and is restricted to statistically significant associations (*P* < .05). Higher scores correspond to higher levels of the indicated construct. As workload, moral distress, and burnout are unfavourable constructs, estimates >1 in the burnout panel reflect greater burnout (less favourable outcomes), whereas estimates >1 in the job satisfaction, engagement, and intention to stay panels indicate more favourable outcomes. The reference line at 1 indicates no association.

**Figure 3.**
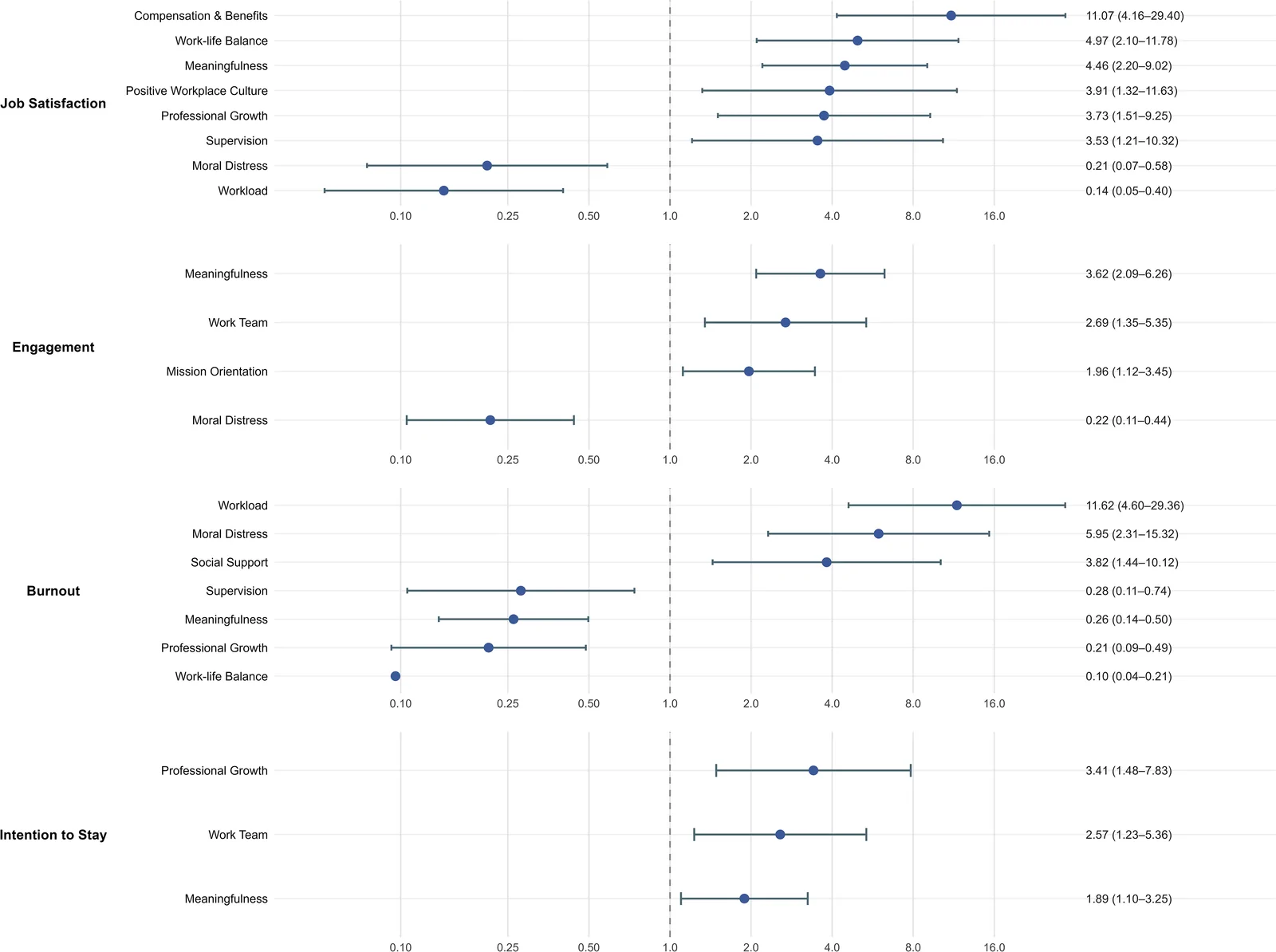
Significant adjusted associations of workforce well-being drivers with job satisfaction, engagement, intention to stay, and burnout Among dentists in HRSA-HCs, 2022-2023. Source: Health Resources and Services Administration (HRSA) Health Centre Workforce Well-being Survey, 2022-2023. Note: Complete model results are presented in Table S3. Multinomial logistic regression models (relative risk ratios [RRRs] and 95% confidence intervals [CIs]) estimated the associations between three-category outcomes and well-being drivers, adjusted for demographic and health centre characteristics for dentists. Binary logistic regression (odds ratios [ORs] and 95% confidence intervals [CIs]) assessed intention to stay. Models were estimated using backward stepwise selection, with variables entered at a significance level of 0.10; age, race and ethnicity, highest education attainment, and marital status were retained in all models. This figure presents adjusted estimates comparing the highest vs lowest level of each well-being driver or outcome, defined as the third vs first tertile or higher vs lower dichotomized level, and is restricted to statistically significant associations (*P* < .05). Higher scores correspond to higher levels of the indicated construct. As workload, moral distress, and burnout are unfavourable constructs, estimates >1 in the burnout panel reflect greater burnout (less favourable outcomes), whereas estimates >1 in the job satisfaction, engagement, and intention to stay panels indicate more favourable outcomes. The reference line at 1 indicates no association.

Detailed adjusted associations between workforce well-being drivers, provider characteristics, health centre characteristics, and well-being outcomes for each oral health provider type are presented in Tables S1-S3. Figures 1-3 display estimated effects comparing the highest vs lowest levels of well-being drivers and outcomes, based on multinominal and binary logistic regression analyses among DAs, DHs, and dentists.

As shown in Figure 1, several workplace factors were consistently associated with well-being outcomes among DAs. More favourable perceptions of work-life balance, professional growth, and compensation and benefits were associated with higher job satisfaction, greater engagement, increased intention to stay, and lower burnout. For example, greater professional growth (RRR = 8.95, 95% CI = 4.25-18.87), compensation and benefits (RRR = 6.63, 95% CI = 3.47-12.64), and work-life balance (RRR = 4.98, 95% CI = 2.75-9.02) were strongly associated with high job satisfaction. Better work-life balance was also strongly associated with lower burnout (RRR = 0.08, 95% CI = 0.04-0.15). In contrast, higher workload and moral distress were consistently associated with less favourable outcomes, particularly higher burnout (workload: RRR = 7.31, 95% CI = 3.78-14.14; moral distress: RRR = 6.63, 95% CI = 3.40-12.95) and lower engagement (moral distress: RRR = 0.23, 95% CI = 0.13-0.40).

Similar patterns were observed among DHs (Figure 2). More favourable perceptions of work-life balance, work meaningfulness, and recognition were associated with better well-being outcomes. For example, high job satisfaction was strongly associated with better work-life balance (RRR = 6.57, 95% CI = 2.34-18.46) and greater recognition (RRR = 5.26, 95% CI = 1.35-20.47). Likewise, high intention to stay was associated with greater recognition (OR = 3.78, 95% CI = 1.22-11.75) and better work-life balance (OR = 3.21, 95% CI = 1.23-8.42). Better work-life balance was also associated with lower burnout (RRR = 0.09, 95% CI = 0.03-0.24). In contrast, greater workload and moral distress were consistently associated with less favourable outcomes, particularly higher burnout (workload: RRR = 11.91, 95% CI = 4.48-31.67) and lower job satisfaction (workload: RRR = 0.16, 95% CI = 0.05-0.46).

Among dentists, similar associations were observed (Figure 3). More favourable perceptions of work-life balance, work meaningfulness, professional growth, supervision, and work team relationships, were associated with better well-being outcomes. For example, high job satisfaction was strongly associated with better work-life balance (RRR = 4.97, 95% CI = 2.10-11.78) and greater work meaningfulness (RRR = 4.46, 95% CI = 2.20-9.02), while high engagement was most strongly associated with work meaningfulness (RRR = 3.62, 95% CI = 2.09-6.26). Intention to stay was positively associated with professional growth (OR = 3.41, 95% CI = 1.48-7.83), and better work-life balance was associated with lower burnout (RRR = 0.10, 95% CI = 0.04-0.21). In contrast, greater workload and moral distress were consistently associated with poorer well-being outcomes, including a higher likelihood of reporting high burnout (workload: RRR = 11.62, 95% CI = 4.60-29.36; moral distress: RRR = 5.95, 95% CI = 2.31-15.32) and low job satisfaction (workload: RRR = 0.14, 95% CI = 0.05-0.40).

Across all provider types, younger staff (<40 years) were consistently less likely to report high job satisfaction and intention to stay, and more likely to report high burnout (Tables S1-S3). Racial and ethnic minority providers were generally less likely to report high burnout except for DHs and were more likely to report high engagement among DHs and dentists. Working in CHC-only or Look-Alike health centres was significantly associated with higher engagement and intention to stay among DHs and dentists. Among dentists, working in smaller or rural health centres was significantly associated with lower engagement and intention to stay. Additional factors, including postgraduate education among DHs and male sex among dentists, were associated with lower engagement or intention to stay.

## Discussion

This study examined workforce well-being among DAs, DHs, and dentists working in HRSA-HCs and identified both shared and provider-specific patterns in the associations between job demands, job resources, and well-being outcomes. Overall, DHs reported the most favourable well-being outcomes, including the highest levels of job satisfaction, engagement, and intention to stay, while burnout was relatively similar across provider types. Positive workplace factors, particularly work-life balance, work meaningfulness, and professional growth were strongly associated with improved well-being outcomes and lower burnout, while workload and moral distress were consistently associated with higher burnout and poorer outcomes across all oral health provider types.

Compensation and benefits, work-life balance, and work meaningfulness were positively associated with job satisfaction across all three provider groups, consistent with previous research. Previous studies have shown that financial compensation, work-life balance, and helping patients improve oral health were the factors most strongly associated with job satisfaction among oral health providers,^20,25,26^ and similar patterns were also observed among HRSA-HC providers more broadly.^5^ This is particularly relevant given that providers in safety-net settings often face constrained resources, lower compensation, and higher patient care demands compared with those in private practice.^27^ Comparable associations have also been observed across the broader healthcare workforce, including among physicians, nurses, and advanced practice providers.^28-31^ These findings suggest that health centres may improve workforce well-being by adopting cross-cutting strategies that support work-life balance, facilitate professional development, underscore the mission-driven nature of oral health care, and ensure competitive compensation and benefits.

Younger oral health providers were also less likely to report high job satisfaction. Longitudinal evidence suggests that job satisfaction may increase with age and organizational transitions, partly reflecting accumulated job rewards.^32^ Selective retention may also contribute, as workers with lower job satisfaction may be more likely to leave, leaving a comparatively more satisfied group among older workers who remain; however, this mechanism cannot be assessed using our cross-sectional data.^33^

Importantly, our study extends this literature by suggesting profession-specific drivers of job satisfaction. Among DAs, professional growth showed the strongest association, consistent with prior literature that limited opportunities for advancement are a major source of dissatisfaction for DAs.^20,34^ Professional growth has also been associated with job satisfaction among NAs.^16,35^ Adequate resources were uniquely important for DAs, suggesting that structural and organizational capacity, including staffing quality, infrastructure, safety systems, and operational processes, may be particularly critical for supporting their job satisfaction. Lack of resources and adequate staffing may increase stress and workload for DAs, which may decrease job satisfaction. Evidence from certified NAs also suggests that adequate resources contribute to job satisfaction.^36^ Improving DA job satisfaction requires adequate operational resources and increasing opportunities for professional advancement by providing training support for expanded-function certifications and developing pathways towards advanced DA roles. Among DHs, recognition and guidance from senior leadership were suggested as additional important contributors, consistent with previous research showing that DHs who feel valued, acknowledged, and adequately supervised report higher job satisfaction.^12,37^ Similar patterns have also been reported among RNs.^38,39^ Among dentists, positive workplace culture, professional growth, and supervisor guidance and engagement were uniquely associated with job satisfaction,^26,40^ consistent with evidence from physicians showing that these factors are associated with greater job satisfaction.^41-43^

Work meaningfulness, work team relationships, and mission orientation were all associated with higher engagement across all three oral health provider types. Although research on engagement among oral health providers is limited, existing studies suggest that job resources related to professional values, patient care, and peer relationships are associated with higher engagement among dentists.^22,41,44^ These findings are also consistent within the broader HRSA-HC context, where mission-driven care for underserved populations may be associated with greater provider engagement.^5^ Across healthcare professions, work meaningfulness, teamwork, and a sense of professional calling have been consistently associated with higher work engagement among nurses and physicians.^38,45,46^ These results suggest that health centres may improve engagement by prioritizing team-based care and formally recognizing provider contributions to patient outcomes and community health.

Notably, work-life balance was uniquely associated with engagement among DAs and DHs, but not dentists, suggesting potential provider-specific differences that warrant further investigation. This distinction may reflect the more structured work environments of DAs and DHs, which afford less scheduling autonomy than dentists, making work-life balance a particularly critical resource for sustaining their engagement. It may also reflect life-stage differences, as DAs and DHs were generally younger and may have greater competing family and caregiving responsibilities. Similar patterns have been observed in the broader healthcare workforce, where work-life balance is positively related to engagement among RNs.^38^ To optimize effectiveness, health centres could tailor engagement strategies by provider type and life stage, incorporating flexible scheduling and family-supportive policies for DAs and DHs.

Our findings suggest that work-life balance, professional growth, and work meaningfulness were important factors associated with lower burnout across most oral health providers. Work-life balance has previously been identified as a significant factor related to lower burnout among DHs and dentists,^6,9,14^ and similar associations have been observed among HRSA-HC providers more broadly.^5^ In contrast, research on associations between professional growth, work meaningfulness, and reduced burnout among oral health providers remains limited, although they are consistent with broader evidence from healthcare professionals showing meaningful work and professional development opportunities may protect nurses and physicians against burnout.^47,48^

We also identified provider-specific factors associated with lower burnout. Among DAs, compensation and benefits, supervision, and supportive health centre processes were associated with lower burnout. Prior research has found that insufficient compensation is related to higher emotional exhaustion among DAs,^49^ while broader healthcare workforce evidence suggests that effective clinical supervision and organizational processes are associated with lower burnout.^50^ Among DHs, adequate resources were suggested to be a particularly protective driver, consistent with previous evidence that low staffing adequacy and insufficient resources are associated with higher burnout among nurses.^17^ Among dentists, supervision was associated with lower burnout, consistent with physician studies showing that higher-quality supervisory leadership is independently associated with lower burnout.^51,52^ These results suggest that health centres may consider provider-specific approaches rather than relying solely on a one-size-fits-all approach. For example, increasing compensation may be more effective for reducing burnout among DAs whereas ensuring adequate resources may be more effective for DHs. Similarly, practice environments that support greater clinical autonomy and opportunities for DHs to practice at the top of their scope may be a promising strategy for enhancing engagement and reducing burnout.

Our findings further suggest that work-life balance, professional growth, and meaningfulness are consistently related to high retention across all oral health providers. Work-life balance and professional growth opportunities have been related to reduced intention to leave among DHs,^12^ and similar associations have been reported among HRSA-HC providers.^5^ These findings are also consistent with broader evidence identifying these three factors as important drivers of retention among nurses and physicians.^18,31,38^ While work-life balance and professional growth have been documented in the DH literature,^12^ their relevance across other oral health provider types, and the role of meaningfulness specifically, has not been previously reported.

However, important differences in retention drivers emerged by provider type. Among DAs, workplace culture, compensation and benefits, and supervision were associated with intention to stay. Negative workplace culture and inadequate compensation have previously been identified as primary factors to DA attrition,^13^ with similar patterns observed among NAs.^16^ The potentially protective role of supervision in supporting DA intention to stay, however, has not been previously reported. Among DHs, recognition was suggested to be particularly influential, consistent with evidence that insufficient rewards and limited promotion opportunities are associated with intention to leave among DH,^12^ and that recognition more broadly is associated with lower intention to leave among RNs.^18^ For dentists, work team relationships were associated with intention to stay, consistent with broader evidence showing that collegial relationships and teamwork are associated with intention to stay among nurses and physicians.^18^ Retention strategies for oral health providers in HRSA-HCs should include flexible scheduling, professional growth opportunities, and recognition for meaningful work while also tailoring strategies to fit provider-specific differences.

Finally, workload and moral distress emerged as important job demands associated with less favourable well-being outcomes across most oral health providers. Workload was strongly associated with higher burnout and poorer job satisfaction, engagement, and intention to stay. This is consistent with previous research identifying high workload as a major contributor to burnout and job dissatisfaction among DHs and dentists,^8,11,14,15,20^ and with broader evidence from HRSA-HC providers and the healthcare workforce showing that workload is associated with burnout, lower job satisfaction, reduced engagement, and higher intention to leave among nurses and physicians.^5,17,18,31,46^ However, its association with all well-being outcomes among DAs have not been previously reported. Similarly, the suggested role of moral distress as a negative driver across multiple well-being outcomes among oral health providers appears to be novel, although it is consistent with broader healthcare workforce evidence linking moral distress to higher burnout and intention to leave.^19^ Addressing these demands requires health centres to combine structural workload mitigation with targeted, role-specific strategies. These may include career pathways for DAs, full-scope utilization for DHs, and dedicated administrative time for dentists to improve well-being and retention.

This study has several limitations. First, cross-sectional data limits the ability to infer causal relationships between well-being drivers and outcomes. Second, although the survey included a large and diverse sample of HRSA-HC staff, participating health centres represented less than 50% of all HRSA-HCs and differed systematically from non-participating centres in size, funding type, and geographic distribution, which may limit the generalizability of findings to all HRSA-HCs. Third, some provider subgroups (eg, DHs and dentists) had smaller sample sizes, which may limit statistical power for certain analyses. Fourth, although the survey included a comprehensive set of individual and health centre characteristics, some information and more detailed variable categories were not publicly available, limiting our ability to explore the effect of provider practice characteristics and socioeconomic factors on well-being outcomes and may have resulted in residual confounding. Last, the analysis relied on self-reported survey data, which may be subject to response bias, particularly for sensitive outcomes such as burnout and intention to leave.

Despite these limitations, the study has several important strengths. It is among the first to examine workforce drivers of well-being among oral health providers in HRSA-HCs using a large, nationally representative dataset. By analysing four well-being outcomes simultaneously, the study provides a more comprehensive understanding than prior work. Furthermore, separate analyses by provider type, incorporating both provider demographics and health centre characteristics; address a key gap by enabling provider-specific insights.

## Conclusion

In conclusion, within the JD-R framework, this study provides novel insights into the well-being of oral health providers at HRSA-HCs by identifying shared and provider-specific associations between job demands, job resources, and well-being outcomes among DAs, DHs, and dentists. Work-life balance, professional growth, and work meaningfulness emerged as common positive factors, while workload and moral distress were suggested as important negative drivers. Provider-specific drivers were also observed. These findings underscore the need for HRSA-HC administrators and policymakers to implement both universal and targeted organizational strategies, such as workload management, professional development, and tailored recognition and supervision programs, to support workforce well-being and retention in safety-net settings. Future research should examine interrelationships among well-being outcomes to inform more targeted interventions.

## Supplementary Material

qxag211_Supplementary_Data

## References

[1] National health center program uniform data system (UDS) awardee data. HRSA Data Warehouse. Accessed February 17, 2026. https://data.hrsa.gov/topics/healthcenters/uds/overview/national/

[2] 2024 health center data. HRSA Data Warehouse. Accessed February 17, 2026. https://data.hrsa.gov/topics/healthcenters/uds/overview/national/table?tableName=Full&year=2024

[3] Workforce projections. HRSA Data Warehouse. December 18, 2025. Accessed March 10, 2026. https://data.hrsa.gov/topics/health-workforce/nchwa/workforce-projections

[4] American Dental Association (ADA). *Economic Outlook and Emerging Issues in Dentistry 4^th^ Quarter, 2025*. Health Policy Institute, ADA; 2025. Accessed February 17, 2026. https://www.ada.org/-/media/project/ada-organization/ada/ada-org/files/resources/research/hpi/q42025_economic_outlook_dentistry_main.pdf?rev=66e851c927104d9da0e4ed6ac61174af&hash=78EB7EB8E9C3C425024278148E525C6E

[5] Jiri T, Mangione T. HRSA Health Center Workforce Well-Being National Data Report: Findings from the 2022 HRSA Health Center Workforce Well-Being Survey. John Snow, Inc.; 2023. https://content.govdelivery.com/accounts/USHHSHRSA/bulletins/39020b0

[6] Calvo JM, Kwatra J, Yansane A, Tokede O, Gorter RC, Kalenderian E. Burnout and work engagement among US dentists. J Patient Saf. 2021;17(5):398–404. 10.1097/PTS.0000000000000355 28671911

[7] *CareQuest Institute for Oral Health. Burnout among Dental Professionals before and during a Public Health Crisis: Causes, Consequences, and next Steps*. Boston, MA: CareQuest Institute; 2022. Accessed March 11, 2026. https://carequest.org/wp-content/uploads/2025/11/CareQuest_Institute_Burnout-Among-Dental-Professionals_8.3.22.pdf

[8] Pollack S, Guenther G, Stubbs B, Patterson D, Frogner B, Skillman S. Characteristics of the Current Dental Assistant and Expanded Function Dental Auxiliary Workforces in Washington State. Center for Health Workforce Studies, University of Washington; 2024.

[9] Bercasio LV, Bercasio LV, Rowe DJ, Rowe DJ, Yansane AI, Yansane AI. Factors associated with burnout among dental hygienists in California. J Dent Hyg. 2020;94(6):40–48. 33376121

[10] Bishop JL, Henderson R, Townsend JA, Kearney RC. Job stressors and their impact on dental hygienists’ job satisfaction and intention to leave practice. Work. 2025;82(1):120–129. 10.1177/10519815251336919 40356512

[11] Knutt A, Boyd LD, Adams JL, Vineyard J. Compassion satisfaction, compassion fatigue, and burnout among dental hygienists in the United States. Am Dent Hyg Assoc. 2022;96(1):34–42. 35190492

[12] Patel BM, Boyd LD, Vineyard J, LaSpina L. Job satisfaction, burnout, and intention to leave among dental hygienists in clinical practice. Am Dent Hyg Assoc. 2021;95(2):28–35. 33875527

[13] *Dental Workforce Shortages: Data to Navigate Today's Labor Market*. ADA Health Policy Institute in collaboration with American Dental Assistants Association, American Dental Hygienists’ Association, Dental Assisting National Board, and IgniteDA; 2022. Accessed June 12, 2025. https://www.ada.org/-/media/project/ada-organization/ada/ada-org/files/resources/research/hpi/dental_workforce_shortages_labor_market.pdf

[14] Singh P, Aulak DS, Mangat SS, Aulak MS. Systematic review: factors contributing to burnout in dentistry. Occup Med (Lond). 2016;66(1):27–31. 10.1093/occmed/kqv119 26443193

[15] Malcolm N, Boyd L, Giblin-Scanlon L, Vineyard J. Occupational stressors of dental hygienists in the United States. Work. 2020;65(3):517–524. 10.3233/wor-203106 32116271

[16] Chang YC, Yeh TF, Lai IJ, Yang CC. Job competency and intention to stay among nursing assistants: the mediating effects of intrinsic and extrinsic job satisfaction. Int J Environ Res Public Health. 2021;18(12):6436. 10.3390/ijerph18126436 PMC8296268 34198623

[17] Dall’Ora C, Ball J, Reinius M, Griffiths P. Burnout in nursing: a theoretical review. Hum Resour Health. 2020;18(1):41. 10.1186/s12960-020-00469-9 PMC7273381 32503559

[18] De Vries N, Boone A, Godderis L, et al The race to retain healthcare workers: a systematic review on factors that impact retention of nurses and physicians in hospitals. Inquiry. 2023;60:00469580231159318. 10.1177/00469580231159318 PMC10014988 36912131

[19] Maunder RG, Heeney ND, Greenberg RA, et al The relationship between moral distress, burnout, and considering leaving a hospital job during the COVID-19 pandemic: a longitudinal survey. BMC Nurs. 2023;22(1):243. 10.1186/s12912-023-01407-5 PMC10369708 37496000

[20] Sasaki N, Pang J, Surdu S, Morrissey RW, Vujicic M, Moore J. Workplace factors associated with job satisfaction among dental hygienists and assistants in the United States. Health Aff Sch. 2025;3(1):qxae147. 10.1093/haschl/qxae147 PMC11726826 39811074

[21] Bates LF, Buehler AM, Boynton JR, Majewski RF, Inglehart MR. Pediatric dentists’ job satisfaction: results of a national survey. Pediatr Dent. 2013;35(4):343–350. 23930634

[22] Hakanen JJ, Bakker AB, Demerouti E. How dentists cope with their job demands and stay engaged: the moderating role of job resources. Eur J Oral Sci. 2005;113(6):479–487. 10.1111/j.1600-0722.2005.00250.x 16324137

[23] Bakker AB, Demerouti E. The job demands-resources model: state of the art. J Manag Psychol. 2007;22(3):309–328. 10.1108/02683940710733115

[24] Health Resources & Services Administration. Health Center Program Compliance Manual. Health Resources & Services Administration. November 2025. Accessed August 2, 2026. https://bphc.hrsa.gov/compliance/compliance-manual/introduction

[25] Lo Sasso AT, Starkel RL, Warren MN, Guay AH, Vujicic M. Practice settings and dentists’ job satisfaction. J Am Dent Assoc. 2015;146(8):600–609. 10.1016/j.adaj.2015.03.001 26227645

[26] Le VNT, Dang MH, Kim JG, Yang YM, Lee DW. Dentist job satisfaction: a systematic review and meta-analysis. Int Dent J. 2021;71(5):369–377. 10.1016/j.identj.2020.12.018 PMC9275337 33612262

[27] Lewis SE, Nocon RS, Tang H, et al Patient-centered medical home characteristics and staff morale in safety net clinics. Arch Intern Med. 2012;172(1):23–31. 10.1001/archinternmed.2011.580 PMC3752653 22232143

[28] Leigh JP, Tancredi DJ, Kravitz RL. Physician career satisfaction within specialties. BMC Health Serv Res. 2009;9(1):166. 10.1186/1472-6963-9-166 PMC2754441 19758454

[29] Kao AC, Jager AJ, Koenig BA, et al Physician perception of pay fairness and its association with work satisfaction, intent to leave practice, and personal health. J Gen Intern Med. 2018;33(6):812–817. 10.1007/s11606-017-4303-8 PMC5975140 29380217

[30] McMurray JE, Williams E, Schwartz MD, et al Physician job satisfaction: developing a model using qualitative data. J Gen Intern Med. 1997;12(11):711–714. 10.1046/j.1525-1497.1997.07145.x PMC1497191 17764023

[31] McHugh MD, Kutney-Lee A, Cimiotti JP, Sloane DM, Aiken LH. Nurses’ widespread job dissatisfaction, burnout, and frustration with health benefits signal problems for patient care. Health Aff (Millwood). 2011;30(2):202–210. 10.1377/hlthaff.2010.0100 PMC3201822 21289340

[32] Dobrow SR, Ganzach Y, Liu Y. Time and job satisfaction: a longitudinal study of the differential roles of age and tenure. J Manag. 2018;44(7):2558–2579. 10.1177/0149206315624962

[33] Rosen J, Stiehl EM, Mittal V, Leana CR. Stayers, leavers, and switchers among certified nursing assistants in nursing homes: a longitudinal investigation of turnover intent, staff retention, and turnover. Gerontologist. 2011;51(5):597–609. 10.1093/geront/gnr025 PMC3179664 21498629

[34] Locker D. Work stress, job satisfaction and emotional well-being among Canadian dental assistants. Community Dent Oral Epidemiol. 1996;24(2):133–137. 10.1111/j.1600-0528.1996.tb00830.x 8654035

[35] Reina AM, Adawi Suker AN, Douglas F, et al Assessing certified nursing assistants’ interest in well-being-oriented continuing education to improve job sustainability and career progression. Gerontol Geriatr Educ. 2026;47(2):237–255. 10.1080/02701960.2025.2500092 40331353

[36] Bryant NS, Cimarolli VR, Falzarano F, Stone R. Organizational factors associated with certified nursing assistants’ job satisfaction during COVID-19. J Appl Gerontol. 2023;42(7):1574–1581. 10.1177/07334648231155017 PMC9908517 36748259

[37] Straub-Bruce LA. Job satisfaction of dental hygienists in Pennsylvania: a quantitative analysis. BMC Health Serv Res. 2025;25(1):1168. 10.1186/s12913-025-13269-5 PMC12403453 40890672

[38] Wei H, Horsley L, Cao Y, et al The associations among nurse work engagement, job satisfaction, quality of care, and intent to leave: a national survey in the United States. Int J Nurs Sci. 2023;10(4):476–484. 10.1016/j.ijnss.2023.09.010 PMC10667320 38020845

[39] López-Ibort N, Cañete-Lairla MA, Gil-Lacruz AI, Gil-Lacruz M, Antoñanzas-Lombarte T. The quality of the supervisor-nurse relationship and its influence on nurses’ job satisfaction. Healthcare (Basel). 2021;9(10):1388. 10.3390/healthcare9101388 PMC8544584 34683067

[40] Vujicic M, Weninger R, Harrison B, Nasseh K, Menezes A. Practice Setting Transitions and Career Satisfaction among New Dentists. Health Policy Institute; 2023.

[41] Burns KEA, Pattani R, Lorens E, Straus SE, Hawker GA. The impact of organizational culture on professional fulfillment and burnout in an academic department of medicine. PLoS One. 2021;16(6):e0252778. 10.1371/journal.pone.0252778 PMC8189486 34106959

[42] Rao S, Ferris TG, Hidrue MK, et al Physician burnout, engagement and career satisfaction in a large academic medical practice. Clin Med Res. 2020;18(1):3–10. 10.3121/cmr.2019.1516 PMC7153796 31959669

[43] Krogstad U, Hofoss D, Veenstra M, Hjortdahl P. Predictors of job satisfaction among doctors, nurses and auxiliaries in Norwegian hospitals: relevance for micro unit culture. Hum Resour Health. 2006;4(1):3. 10.1186/1478-4491-4-3 PMC1397856 16483384

[44] Gorter RC, Te Brake HJHM, Hoogstraten J, Eijkman MAJ. Positive engagement and job resources in dental practice. Community Dent Oral Epidemiol. 2008;36(1):47–54. 10.1111/j.1600-0528.2007.00350.x 18205640

[45] Prenestini A, Palumbo R, Grilli R, Lega F. Exploring physician engagement in health care organizations: a scoping review. BMC Health Serv Res. 2023;23(1):1029. 10.1186/s12913-023-09935-1 PMC10521513 37749568

[46] Montgomery A, Spânu F, Băban A, Panagopoulou E. Job demands, burnout, and engagement among nurses: a multi-level analysis of ORCAB data investigating the moderating effect of teamwork. Burn Res. 2015;2(2-3):71–79. 10.1016/j.burn.2015.06.001 PMC4710673 26877971

[47] Correia I, Almeida AE. Organizational justice, professional identification, empathy, and meaningful work during COVID-19 pandemic: are they burnout protectors in physicians and nurses? Front Psychol. 2020;11:566139. 10.3389/fpsyg.2020.566139 PMC7759469 33362629

[48] Razai MS, Kooner P, Majeed A. Strategies and interventions to improve healthcare professionals’ well-being and reduce burnout. J Prim Care Community Health. 2023;14:21501319231178641. 10.1177/21501319231178641 PMC10233581 37246649

[49] Uziel N, Meyerson J, Birenzweig Y, Eli I. Professional burnout and work stress among Israeli dental assistants. Psychol Health Med. 2019;24(1):59–67. 10.1080/13548506.2018.1475679 29768027

[50] Martin P, Lizarondo L, Kumar S, Snowdon D. Impact of clinical supervision on healthcare organisational outcomes: a mixed methods systematic review. PLoS One. 2021;16(11):e0260156. 10.1371/journal.pone.0260156 PMC8604366 34797897

[51] Dyrbye LN, Major-Elechi B, Hays JT, Fraser CH, Buskirk SJ, West CP. Physicians’ ratings of their supervisor's leadership behaviors and their subsequent burnout and satisfaction: a longitudinal study. Mayo Clin Proc. 2021;96(10):2598–2605. 10.1016/j.mayocp.2021.01.035 34538425

[52] Shanafelt TD, Gorringe G, Menaker R, et al Impact of organizational leadership on physician burnout and satisfaction. Mayo Clin Proc. 2015;90(4):432–440. 10.1016/j.mayocp.2015.01.012 25796117

