# Supplementary material for: Workplace indicators and well-being among oral health professionals in HRSA-funded health centres
Source: Health Aff Sch. 2026 Aug 13;4(8):qxag211. doi: 10.1093/haschl/qxag211 (PMC13594666; doi:10.1093/haschl/qxag211)
Supplement: Supplementary file 1 [file qxag211_Supplementary_Data.zip › Supplemental tables 1-3.docx]

Supplemental Table 1. Adjusted Association Between Workforce Well-being Drivers and Four Outcomes among Dental Assistants in HRSA-HCs, 2022-2023

|  | **Dental Assistants (n=1,435)** | | | | | | | |
| --- | --- | --- | --- | --- | --- | --- | --- | --- |
|  | **Job Satisfaction^5^** | | **Engagement^5^** | | | **Burnout^5^** | | **Intent to Stay^5^** |
|  | **Middle vs Low**  **(T2 vs. T1)** | **High vs Low**  **(T3 vs. T1)** | | **Middle vs Low**  **(T2 vs. T1)** | **High vs Low**  **(T3 vs. T1)** | **Middle vs Low**  **(T2 vs. T1)** | **High vs Low**  **(T3 vs. T1)** | **High vs Low**  **(M2 vs. M1)** |
|  | **RRR**  **(95% CI)*** | **RRR**  **(95% CI)*** | | **RRR**  **(95% CI)*** | **RRR**  **(95% CI)*** | **RRR**  **(95% CI)*** | **RRR**  **(95% CI)*** | **OR**  **(95% CI)*** |
| **Well-being Drivers^5^** |  |  | |  |  |  |  |  |
| Work Team |  |  | |  |  |  |  |  |
| Low (T1) | ref | | | | | | | |
| Middle (T2) |  |  | | 2.01 (1.32, 3.07)** | 1.53 (0.96, 2.43) |  |  |  |
| High (T3) |  |  | | 3.30 (1.92, 5.68)*** | 2.74 (1.58, 4.77)*** | |  |  |
| Supervision |  |  | |  |  |  |  |  |
| Low (T1) | ref | | | | | | | |
| Middle (T2) |  |  | |  |  | 0.74 (0.46, 1.20) | 0.74 (0.42, 1.31) | 1.21 (0.81, 1.82) |
| High (T3) |  |  | |  |  | 0.46 (0.28, 0.77)** | 0.33 (0.18, 0.62)** | 1.74 (1.11, 2.73)* |
| Leadership |  |  | |  |  |  |  |  |
| Low (T1) | ref | | | | | | | |
| Middle (T2) | 1.97 (1.27, 3.06)** | 2.87 (1.59, 5.17)*** | | |  |  |  |  |
| High (T3) | 2.24 (1.15, 4.36)* | 3.42 (1.58, 7.39)** | | |  |  |  |  |
| Positive Workplace Culture |  |  | |  |  |  |  |  |
| Low (T1) | ref | | | | | | | |
| Middle (T2) |  |  | |  |  |  |  | 2.02 (1.35, 3.01)** |
| High (T3) |  |  | |  |  |  |  | 3.24 (1.90, 5.54)*** |
| Social Support |  |  | |  |  |  |  |  |
| Low (T1) | ref | | | | | | | |
| Middle (T2) | 1.51 (0.96, 2.39) | 1.13 (0.61, 2.09) | | 1.54 (0.99, 2.38) | 1.75 (1.08, 2.84)* |  |  |  |
| High (T3) | 1.10 (0.62, 1.97) | 1.82 (0.91, 3.62) | | 0.76 (0.43, 1.32) | 1.16 (0.65, 2.06) |  |  |  |
| Recognition |  |  | |  |  |  |  |  |
| Low (T1) | ref | | | | | | | |
| Middle (T2) | 2.01 (1.29, 3.12)** | 1.61 (0.89, 2.89) | |  |  | 0.89 (0.55, 1.45) | 0.43 (0.25, 0.76)** | |
| High (T3) | 1.69 (0.87, 3.28) | 1.02 (0.48, 2.18) | |  |  | 1.19 (0.66, 2.15) | 0.78 (0.38, 1.59) |  |
| Supportive Health Center Processes |  |  | |  |  |  |  |  |
| Low (T1) | ref | | | | | | | |
| Middle (T2) |  |  | | 0.82 (0.56, 1.21) | 0.65 (0.42, 0.99)* | 1.15 (0.74, 1.78) | 1.06 (0.63, 1.79) |  |
| High (T3) |  |  | | 0.68 (0.40, 1.15) | 1.31 (0.78, 2.19) | 0.55 (0.34, 0.89)* | 0.49 (0.26, 0.91)* |  |
| Training Provided |  |  | |  |  |  |  |  |
| Low (T1) | ref | | | | | | | |
| Middle (T2) |  |  | |  |  |  |  |  |
| High (T3) |  |  | |  |  |  |  |  |
| Adequate Resources |  |  | |  |  |  |  |  |
| Low (T1) | ref | | | | | | | |
| Middle (T2) | 1.81 (1.15, 2.84)* | 2.27 (1.24, 4.15)** | | 1.78 (1.16, 2.75)** | 1.20 (0.75, 1.95) |  |  |  |
| High (T3) | 1.18 (0.63, 2.21) | 3.45 (1.70, 7.01)** | | 0.99 (0.54, 1.78) | 0.83 (0.46, 1.52) |  |  |  |
| Mission Orientation |  |  | |  |  |  |  |  |
| Low (M1) | ref | | | | | | | |
| High (M2) |  |  | | 1.35 (0.87, 2.11) | 2.07 (1.31, 3.27)** | |  |  |
| Meaningfulness |  |  | |  |  |  |  |  |
| Low (M1) | ref | | | | | | | |
| High (M2) | 1.07 (0.71, 1.62) | 3.80 (2.32, 6.21)*** | | 1.15 (0.75, 1.77) | 2.89 (1.86, 4.49)*** | |  |  |
| Compensation & Benefits |  |  | |  |  |  |  |  |
| Low (T1) | ref | | | | | | | |
| Middle (T2) | 1.33 (0.88, 2.00) | 1.82 (1.06, 3.14)* | |  |  | 0.67 (0.42, 1.06) | 0.45 (0.26, 0.77)** | 1.81 (1.25, 2.61)** |
| High (T3) | 2.44 (1.38, 4.31)** | 6.63 (3.47, 12.64)*** | | |  | 0.59 (0.36, 0.95)* | 0.29 (0.15, 0.54)*** | 2.19 (1.34, 3.57)** |
| Professional Growth |  |  | |  |  |  |  |  |
| Low (T1) | ref | | | | | | | |
| Middle (T2) | 2.82 (1.87, 4.27)*** | 2.19 (1.24, 3.88)** | | 1.29 (0.85, 1.94) | 0.87 (0.56, 1.36) | 1.07 (0.66, 1.73) | 0.67 (0.39, 1.17) | 2.34 (1.60, 3.42)*** |
| High (T3) | 3.76 (1.93, 7.35)*** | 8.95 (4.25, 18.87)*** | | 1.35 (0.74, 2.46) | 1.50 (0.83, 2.71) | 0.37 (0.21, 0.67)** | 0.24 (0.11, 0.49)*** | 2.00 (1.16, 3.46)* |
| Workload |  |  | |  |  |  |  |  |
| Low (T1) | ref | | | | | | | |
| Middle (T2) | 0.89 (0.56, 1.42) | 0.66 (0.39, 1.13) | | 1.08 (0.70, 1.68) | 0.67 (0.42, 1.07) | 2.26 (1.51, 3.37)*** | 2.55 (1.46, 4.44)** | |
| High (T3) | 0.49 (0.30, 0.81)** | 0.31 (0.16, 0.58)*** | | 0.95 (0.57, 1.57) | 1.23 (0.72, 2.08) | 3.05 (1.73, 5.38)*** | 7.31 (3.78, 14.14)*** | |
| Work-life Balance |  |  | |  |  |  |  |  |
| Low (T1) | ref | | | | | | | |
| Middle (T2) | 1.65 (1.09, 2.50)* | 1.64 (0.96, 2.80) | | 2.55 (1.71, 3.82)*** | 2.19 (1.41, 3.40)*** | 0.38 (0.24, 0.60)*** | 0.15 (0.09, 0.27)*** | 1.41 (0.98, 2.02) |
| High (T3) | 2.50 (1.49, 4.18)** | 4.98 (2.75, 9.02)*** | | 1.96 (1.19, 3.23)** | 2.38 (1.44, 3.95)** | 0.15 (0.09, 0.25)*** | 0.08 (0.04, 0.15)*** | 2.48 (1.53, 3.99)*** |
| Moral Distress |  |  | |  |  |  |  |  |
| Low (T1) | ref | | | | | | | |
| Middle (T2) |  |  | | 0.33 (0.20, 0.54)*** | 0.31 (0.19, 0.51)*** | 1.59 (1.06, 2.38)* | 2.10 (1.18, 3.72)* |  |
| High (T3) |  |  | | 0.20 (0.11, 0.34)*** | 0.23 (0.13, 0.40)*** | 2.08 (1.20, 3.63)* | 6.63 (3.40, 12.95)*** | |
| **Variables** |  |  | |  |  |  |  |  |
| Age |  |  | |  |  |  |  |  |
| Under 40 | 0.63 (0.41, 0.95)* | 0.46 (0.28, 0.77)** | | 0.58 (0.40, 0.85)** | 0.61 (0.41, 0.91)* | 1.59 (1.06, 2.38)* | 3.92 (2.35, 6.53)*** | 0.36 (0.25, 0.53)*** |
| 40 and older | ref | | | | | | | |
| Gender |  |  | |  |  |  |  |  |
| Male |  |  | |  |  | 1.00 (0.46, 2.19) | 0.33 (0.11, 0.98)* |  |
| Female/Other^1^ | ref | | | | | | | |
| Race/Ethnicity |  |  | |  |  |  |  |  |
| White Non-Hispanic | ref | | | | | | | |
| All Other Race^2^ Ethnicities | 0.64 (0.44, 0.95)* | 0.69 (0.42, 1.11) | | 1.32 (0.92, 1.90) | 1.18 (0.81, 1.73) | 0.49 (0.32, 0.73)** | 0.27 (0.17, 0.45)*** | 0.79 (0.57, 1.10) |
| Highest Education |  |  | |  |  |  |  |  |
| High school or less | ref | | | | | | | |
| Certification or Some College | 0.82 (0.45, 1.46) | 0.78 (0.39, 1.55) | | 1.19 (0.72, 1.97) | 1.65 (0.93, 2.91) | 0.67 (0.39, 1.14) | 0.77 (0.39, 1.54) | 1.04 (0.64, 1.71) |
| Associate or Bachelor's Degree or Post Graduate Degree^3^ | 0.85 (0.45, 1.60) | 0.63 (0.29, 1.34) | | 0.82 (0.47, 1.43) | 1.18 (0.64, 2.19) | 0.72 (0.40, 1.30) | 0.92 (0.44, 1.94) | 1.05 (0.61, 1.81) |
| Marital Status |  |  | |  |  |  |  |  |
| Married | 0.65 (0.42, 1.01) | 0.68 (0.39, 1.18) | | 0.99 (0.66, 1.48) | 1.16 (0.76, 1.78) | 1.13 (0.72, 1.76) | 1.45 (0.85, 2.47) | 0.67 (0.47, 0.97)* |
| Never Married | ref | | | | | | | |
| Previously Married/Separated | 0.56 (0.32, 0.98)* | 0.68 (0.35, 1.32) | | 1.05 (0.63, 1.75) | 0.92 (0.54, 1.59) | 0.94 (0.55, 1.58) | 1.11 (0.57, 2.15) | 0.70 (0.43, 1.16) |
| Urban/Rural |  |  | |  |  |  |  |  |
| Rural |  |  | |  |  |  |  |  |
| Urban | ref | | | | | | | |
| Health Center Size |  |  | |  |  |  |  |  |
| Small |  |  | |  |  |  |  |  |
| Large | ref | | | | | | | |
| Health Center Funding |  |  | |  |  |  |  |  |
| CHC only or Look Alike^4^ |  |  | |  |  | 0.90 (0.61, 1.32) | 0.62 (0.39, 0.99)* |  |
| Multiply funded | ref | | | | | | | |
| HRSA Region |  |  | |  |  |  |  |  |
| Region 1 (CT, ME, MA, NH, RI, VT) |  |  | |  |  |  |  |  |
| Region 2 (NJ, NY, PR, Virgin Islands) |  |  | | 1.87 (0.88, 3.98) | 3.31 (1.58, 6.90)** | |  |  |
| Region 3 (DE, DC, MD, PA, VA, WV) |  |  | |  |  |  |  |  |
| Region 4 (AL, FL, GA, KY, MS, NC, SC, TN) |  |  | |  |  |  |  | 0.58 (0.32, 1.06) |
| Region 5 (IL, IN, MI, MN, OH, WI) |  |  | |  |  |  |  |  |
| Region 6 (AR, LA, NM, OK, TX) |  |  | |  |  |  |  |  |
| Region 7 (IA, MO, NE, KS) |  |  | |  |  |  |  |  |
| Region 8 (CO, MT, ND, SD, UT, WY) |  |  | |  |  |  |  |  |
| Region 9 (AZ, CA, HI, NV, American Samoa, Guam, Northern Mariana Islands, Federated States of Micronesia, Marshall Islands, Palau) | ref | | | | | | | |
| Region 10 (AK, ID, OR, WA) |  |  | |  |  |  |  |  |

Source: Health Resources and Services Administration (HRSA) Health Center Workforce Well-being Survey, 2022-2023.

Note: ^1^ Female/Other, female, female-to-male (FTM)/transgender male/trans man, male-to=female (MTF)/transgender female/trans women, genderqueer, neither exclusively male or female, something else, and don’t know/not sure based on gender identity.

^2^ All Other Race Ethnicities, Hispanic, Black Non-Hispanic, and Other Non-Hispanic.

^3^ Associate or Bachelor's Degree or Post Graduate Degree, Associate’s degree (e.g., AA, AS), Bachelor’s degree (e.g., BA, BS), Master’s degree (e.g., MA, MS, MBA), Doctoral/Professional degree (e.g., MD/DO, DMD/DDS, PhD).

^4^ CHC only or Look Alike, Community Health Center (CHC) Only.

^5^ Survey items were assessed on a 6-point Likert scale ranging from (1) strongly disagree to (6) strongly agree and reverse scored as needed so that higher composite scores represented greater levels of the construct named. Higher burnout, workload, and moral distress scores indicate less favorable well-being outcomes or workplace conditions. T1, T2, and T3 denote the low, middle, and high tertiles, respectively; M1 and M2 denote the low and high dichotomized categories, respectively.

Supplemental Table 2. Adjusted Association Between Workforce Well-being Drivers and Four Outcomes among Dental Hygienists in HRSA-HCs, 2022-2023

|  | **Dental Hygienists (n=564)** | | | | | | | | | | | | | |  |  |  |  |  |  |
| --- | --- | --- | --- | --- | --- | --- | --- | --- | --- | --- | --- | --- | --- | --- | --- | --- | --- | --- | --- | --- |
|  | **Job Satisfaction^6^** | | | | **Engagement^6^** | | | | | **Burnout^6^** | | | | **Intent to Stay^6^** |  |  |  |  |  |  |
|  | **Middle vs Low**  **(T2 vs. T1)** | | **High vs Low**  **(T3 vs. T1)** | | | **Middle vs Low**  **(T2 vs. T1)** | | **High vs Low**  **(T3 vs. T1)** | | **Middle vs Low**  **(T2 vs. T1)** | | **High vs Low**  **(T3 vs. T1)** | | **High vs Low**  **(M2 vs. M1)** |  |  |  |  |  |  |
|  | **RRR**  **(95% CI)*** | | **RRR**  **(95% CI)*** | | | **RRR**  **(95% CI)*** | | **RRR**  **(95% CI)*** | | **RRR**  **(95% CI)*** | | **RRR**  **(95% CI)*** | | **OR**  **(95% CI)*** |  |  |  |  |  |  |
| **Well-being Drivers^6^** |  | |  | | |  | |  | |  | |  | |  |  |  |  |  |  |  |
| Work Team |  | |  | | |  | |  | |  | |  | |  |  |  |  |  |  |  |
| Low (T1) | ref | | | | | | | | | | | | | |  |  |  |  |  |  |
| Middle (T2) |  | |  | | | 1.29 (0.55, 3.00) | | 1.91 (1.00, 3.62)* | |  | |  | |  |  |  |  |  |  |  |
| High (T3) |  | |  | | | 0.56 (0.19, 1.66) | | 3.61 (1.86, 7.00)*** | | | |  | |  |  |  |  |  |  |  |
| Supervision |  | |  | | |  | |  | |  | |  | |  |  |  |  |  |  |  |
| Low (T1) | ref | | | | | | | | | | | | | |  |  |  |  |  |  |
| Middle (T2) | 3.00 (1.52, 5.93)** | | 3.23 (1.22, 8.56)* | | |  | |  | | 1.05 (0.52, 2.13) | | 0.36 (0.16, 0.80)* | |  |  |  |  |  |  |  |
| High (T3) | 2.35 (0.98, 5.66) | | 2.03 (0.62, 6.61) | | |  | |  | | 0.58 (0.28, 1.22) | | 0.48 (0.20, 1.13) | |  |  |  |  |  |  |  |
| Leadership |  | |  | | |  | |  | |  | |  | |  |  |  |  |  |  |  |
| Low (T1) | ref | | | | | | | | | | | | | |  |  |  |  |  |  |
| Middle (T2) | 1.14 (0.56, 2.31) | | 1.78 (0.61, 5.20) | | | | |  | |  | |  | |  |  |  |  |  |  |  |
| High (T3) | 1.02 (0.33, 3.09) | | 4.30 (1.08, 17.07)* | | | | |  | |  | |  | |  |  |  |  |  |  |  |
| Positive Workplace Culture |  | |  | | |  | |  | |  | |  | |  |  |  |  |  |  |  |
| Low (T1) | ref | | | | | | | | | | | | | |  |  |  |  |  |  |
| Middle (T2) | 1.88 (0.95, 3.75) | | 0.53 (0.19, 1.52) | | |  | |  | |  | |  | |  |  |  |  |  |  |  |
| High (T3) | 0.79 (0.30, 2.10) | | 0.31 (0.09, 1.10) | | |  | |  | |  | |  | |  |  |  |  |  |  |  |
| Social Support |  | |  | | |  | |  | |  | |  | |  |  |  |  |  |  |  |
| Low (T1) | ref | | | | | | | | | | | | | |  |  |  |  |  |  |
| Middle (T2) |  | |  | | |  | |  | |  | |  | |  |  |  |  |  |  |  |
| High (T3) |  | |  | | |  | |  | |  | |  | |  |  |  |  |  |  |  |
| Recognition |  | |  | | |  | |  | |  | |  | |  |  |  |  |  |  |  |
| Low (T1) | ref | | | | | | | | | | | | | |  |  |  |  |  |  |
| Middle (T2) | 1.86 (0.93, 3.73) | | 1.84 (0.63, 5.39) | | |  | |  | |  | |  | | 1.92 (0.98, 3.75) |  |  |  |  |  |  |
| High (T3) | 3.41 (1.23, 9.47)* | | 5.26 (1.35, 20.47)* | | |  | |  | |  | |  | | 3.78 (1.22, 11.75)* |  |  |  |  |  |  |
| Supportive Health Center Processes |  | |  | | |  | |  | |  | |  | |  |  |  |  |  |  |  |
| Low (T1) | ref | | | | | | | | | | | | | |  |  |  |  |  |  |
| Middle (T2) |  | |  | | |  | |  | |  | |  | |  |  |  |  |  |  |  |
| High (T3) |  | |  | | |  | |  | |  | |  | |  |  |  |  |  |  |  |
| Training Provided |  | |  | | |  | |  | |  | |  | |  |  |  |  |  |  |  |
| Low (T1) | ref | | | | | | | | | | | | | |  |  |  |  |  |  |
| Middle (T2) |  | |  | | | 0.46 (0.16, 1.31) | | 1.31 (0.71, 2.41) | |  | |  | | 2.02 (0.99, 4.10) |  |  |  |  |  |  |
| High (T3) |  | |  | | | 2.96 (1.12, 7.81)* | | 1.59 (0.80, 3.16) | |  | |  | | 2.64 (0.94, 7.40) |  |  |  |  |  |  |
| Adequate Resources |  | |  | | |  | |  | |  | |  | |  |  |  |  |  |  |  |
| Low (T1) | ref | | | | | | | | | | | | | |  |  |  |  |  |  |
| Middle (T2) | 2.11 (1.11, 4.01)* | | 1.64 (0.64, 4.16) | | |  | |  | | 0.52 (0.26, 1.04) | | 0.16 (0.07, 0.37)*** | | 3.28 (1.52, 7.08)** |  |  |  |  |  |  |
| High (T3) | 1.44 (0.55, 3.73) | | 2.48 (0.78, 7.86) | | |  | |  | | 0.33 (0.15, 0.73)** | | 0.08 (0.03, 0.24)*** | | 3.01 (0.93, 9.75) |  |  |  |  |  |  |
| Mission Orientation |  | |  | | |  | |  | |  | |  | |  |  |  |  |  |  |  |
| Low (M1) | ref | | | | | | | | | | | | | |  |  |  |  |  |  |
| High (M2) |  | |  | | | 1.88 (0.78, 4.55) | | 3.28 (1.87, 5.76)*** | | | |  | | 0.38 (0.19, 0.79)** |  |  |  |  |  |  |
| Meaningfulness |  | |  | | |  | |  | |  | |  | |  |  |  |  |  |  |  |
| Low (M1) | ref | | | | | | | | | | | | | |  |  |  |  |  |  |
| High (M2) | 1.26 (0.70, 2.26) | | 3.41 (1.62, 7.20)** | | | 1.66 (0.76, 3.64) | | 2.69 (1.60, 4.54)*** | | 0.76 (0.44, 1.32) | | 0.39 (0.19, 0.78)** | | 2.14 (1.07, 4.27)* |  |  |  |  |  |  |
| Compensation & Benefits |  | |  | | |  | |  | |  | |  | |  |  |  |  |  |  |  |
| Low (T1) | ref | | | | | | | | | | | | | |  |  |  |  |  |  |
| Middle (T2) | 1.28 (0.67, 2.44) | | 3.46 (1.45, 8.27)** | | |  | |  | | 0.58 (0.30, 1.12) | | 0.54 (0.25, 1.20) | |  |  |  |  |  |  |  |
| High (T3) | 2.17 (1.04, 4.51)* | | 5.84 (2.31, 14.72)*** | | | | |  | | 0.28 (0.15, 0.54)*** | | 0.56 (0.25, 1.28) | |  |  |  |  |  |  |  |
| Professional Growth |  | |  | | |  | |  | |  | |  | |  |  |  |  |  |  |  |
| Low (T1) | ref | | | | | | | | | | | | | |  |  |  |  |  |  |
| Middle (T2) |  | |  | | |  | |  | |  | |  | |  |  |  |  |  |  |  |
| High (T3) |  | |  | | |  | |  | |  | |  | |  |  |  |  |  |  |  |
| Workload |  | |  | | |  | |  | |  | |  | |  |  |  |  |  |  |  |
| Low (T1) | ref | | | | | | | | | | | | | |  |  |  |  |  |  |
| Middle (T2) | 0.94 (0.45, 1.95) | | 0.30 (0.13, 0.70)** | | |  | |  | | 1.95 (1.09, 3.50)* | | 2.81 (1.22, 6.46)* | | |  |  |  |  |  |  |
| High (T3) | 0.65 (0.29, 1.45) | | 0.16 (0.05, 0.46)** | | |  | |  | | 3.29 (1.41, 7.69)** | | 11.91 (4.48, 31.67)*** | | |  |  |  |  |  |  |
| Work-life Balance |  | |  | | |  | |  | |  | |  | |  |  |  |  |  |  |  |
| Low (T1) | ref | | | | | | | | | | | | | |  |  |  |  |  |  |
| Middle (T2) | 1.45 (0.79, 2.66) | | 1.31 (0.56, 3.06) | | | 0.58 (0.24, 1.37) | | 0.63 (0.35, 1.13) | | 0.42 (0.21, 0.83)* | | 0.21 (0.10, 0.46)*** | | 1.84 (0.98, 3.45) |  |  |  |  |  |  |
| High (T3) | 1.62 (0.69, 3.82) | | 6.57 (2.34, 18.46)*** | | | 1.08 (0.39, 3.02) | | 1.93 (1.01, 3.69)* | | 0.29 (0.13, 0.64)** | | 0.09 (0.03, 0.24)*** | | 3.21 (1.23, 8.42)* |  |  |  |  |  |  |
| Moral Distress |  | |  | | |  | |  | |  | |  | |  |  |  |  |  |  |  |
| Low (T1) | ref | | | | | | | | | | | | | |  |  |  |  |  |  |
| Middle (T2) | 0.44 (0.21, 0.93)* | | 0.42 (0.17, 0.99)* | | |  | |  | |  | |  | |  |  |  |  |  |  |  |
| High (T3) | 0.27 (0.12, 0.62)** | | 0.26 (0.09, 0.77)* | | |  | |  | |  | |  | | |  |  |  |  |  |  |
| **Variables** |  | |  | | |  | |  | |  | |  | |  |  |  |  |  |  |  |
| Age |  | |  | | |  | |  | |  | |  | |  |  |  |  |  |  |  |
| Under 40 | 0.51 (0.28, 0.93)* | | 0.44 (0.20, 0.94)* | | | 0.57 (0.26, 1.26) | | 1.02 (0.61, 1.72) | | 1.39 (0.80, 2.44) | | 1.95 (0.98, 3.88) | | 0.41 (0.22, 0.77)** |  |  |  |  |  |  |
| 40 and older | ref | | | | | | | | | | | | | |  |  |  |  |  |  |
| Gender |  | |  | | |  | |  | |  | |  | |  |  |  |  |  |  |  |
| Male |  | |  | | |  | |  | |  | |  | | 0.21 (0.04, 1.06) |  |  |  |  |  |  |
| Female/Other^1^ | ref | | | | | | | | | | | | | |  |  |  |  |  |  |
| Race/Ethnicity |  | |  | | |  | |  | |  | |  | |  |  |  |  |  |  |  |
| White Non-Hispanic | ref | | | | | | | | | | | | | |  |  |  |  |  |  |
| All Other Race^2^ Ethnicities | 0.72 (0.38, 1.36) | | 0.53 (0.24, 1.16) | | | 1.64 (0.73, 3.69) | | 1.80 (1.04, 3.09)* | | 0.71 (0.39, 1.26) | | 1.08 (0.52, 2.22) | | 0.41 (0.22, 0.76)** |  |  |  |  |  |  |
| Highest Education |  | |  | | |  | |  | |  | |  | |  |  |  |  |  |  |  |
| Associate or Bachelor's Degree^3^ | ref | | | | | | | | | | | | | |  |  |  |  |  |  |
| Post Graduate Degree^4^ | 1.32 (0.36, 4.82) | | 3.17 (0.64, 15.68) | | |  | | 1.80 (0.60, 5.39) | | 0.45 (0.13, 1.51) | | 0.61 (0.15, 2.55) | | 0.18 (0.06, 0.61)** |  |  |  |  |  |  |
| Marital Status |  | |  | | |  | |  | |  | |  | |  |  |  |  |  |  |  |
| Married | 0.76 (0.31, 1.85) | | 1.02 (0.33, 3.18) | | | 1.36 (0.40, 4.62) | | 2.10 (1.00, 4.40)* | | 1.11 (0.48, 2.54) | | 1.05 (0.38, 2.88) | | 0.67 (0.30, 1.51) |  |  |  |  |  |  |
| Never Married | ref | | | | | | | | | | | | | |  |  |  |  |  |  |
| Previously Married/Separated | 0.94 (0.44, 2.02) | | 1.03 (0.41, 2.61) | | | 1.08 (0.36, 3.19) | | 2.49 (1.29, 4.81)** | | 0.79 (0.39, 1.57) | | 0.77 (0.32, 1.86) | | 1.43 (0.55, 3.70) |  |  |  |  |  |  |
| Urban/Rural |  | |  | | |  | |  | |  | |  | |  |  |  |  |  |  |  |
| Rural |  |  | |  | | |  | |  | |  | |  | |  |  |  |  |  |  |
| Urban | ref | | | | | | | | | | | | | |  |  |  |  |  |  |
| Health Center Size |  | |  | | |  | |  | |  | |  | |  |  |  |  |  |  |  |
| Small | 0.59 (0.31, 1.12) | | 0.40 (0.17, 0.94)* | | |  | |  | |  | |  | |  |  |  |  |  |  |  |
| Large | ref | | | | | | | | | | | | | |  |  |  |  |  |  |
| Health Center Funding |  | |  | | |  | |  | |  | |  | |  |  |  |  |  |  |  |
| CHC only or Look Alike^5^ |  | |  | | | 1.67 (0.73, 3.81) | | 1.77 (1.04, 3.01)* | |  | |  | |  |  |  |  |  |  |  |
| Multiply funded | ref | | | | | | | | | | | | | |  |  |  |  |  |  |
| HRSA Region |  | |  | | |  | |  | |  | |  | |  |  |  |  |  |  |  |
| Region 1 (CT, ME, MA, NH, RI, VT) |  | |  | | |  | |  | | 2.73 (1.11, 6.72)* | | 2.19 (0.71, 6.75) | |  |  |  |  |  |  |  |
| Region 2 (NJ, NY, PR, Virgin Islands) |  | |  | | |  | |  | | | |  | |  |  |  |  |  |  |  |
| Region 3 (DE, DC, MD, PA, VA, WV) |  | |  | | | 0.41 (0.09, 1.94) | | 0.22 (0.08, 0.59)** | |  | |  | | 0.34 (0.14, 0.81)* |  |  |  |  |  |  |
| Region 4 (AL, FL, GA, KY, MS, NC, SC, TN) |  | |  | | |  | |  | |  | |  | |  |  |  |  |  |  |  |
| Region 5 (IL, IN, MI, MN, OH, WI) |  | |  | | |  | |  | |  | |  | | 0.51 (0.25, 1.05) |  |  |  |  |  |  |
| Region 6 (AR, LA, NM, OK, TX) |  | |  | | |  | |  | |  | |  | |  |  |  |  |  |  |  |
| Region 7 (IA, MO, NE, KS) |  | |  | | | 1.47 (0.56, 3.84) | | 0.41 (0.18, 0.94)* | |  | |  | |  |  |  |  |  |  |  |
| Region 8 (CO, MT, ND, SD, UT, WY) |  | |  | | |  | |  | |  | |  | | 0.15 (0.04, 0.49)** |  |  |  |  |  |  |
| Region 9 (AZ, CA, HI, NV, American Samoa, Guam, Northern Mariana Islands, Federated States of Micronesia, Marshall Islands, Palau) | ref | | | | | | | | | | | | | |  |  |  |  |  |  |
| Region 10 (AK, ID, OR, WA) |  | |  | | | 0.18 (0.02, 1.41) | | 0.35 (0.15, 0.84)* | |  | |  | |  |  |  |  |  |  |  |

Source: Health Resources and Services Administration (HRSA) Health Center Workforce Well-being Survey, 2022-2023.

Note: ^1^ Female/Other, female, female-to-male (FTM)/transgender male/trans man, male-to=female (MTF)/transgender female/trans women, genderqueer, neither exclusively male or female, something else, and don’t know/not sure based on gender identity.

^2^ All Other Race Ethnicities, Hispanic, Black Non-Hispanic, and Other Non-Hispanic.

^3^ Associate or Bachelor's Degree, Associate’s degree (e.g., AA, AS), Bachelor’s degree (e.g., BA, BS), Master’s degree (e.g., MA, MS, MBA).

^4^ Post Graduate Degree, Doctoral/Professional degree (e.g., MD/DO, DMD/DDS, PhD).

^5^ CHC only or Look Alike, Community Health Center (CHC) Only.

^6^ Survey items were assessed on a 6-point Likert scale ranging from (1) strongly disagree to (6) strongly agree and reverse scored as needed so that higher composite scores represented greater levels of the construct named. Higher burnout, workload, and moral distress scores indicate less favorable well-being outcomes or workplace conditions. T1, T2, and T3 denote the low, middle, and high tertiles, respectively; M1 and M2 denote the low and high dichotomized categories, respectively.

Supplemental Table 3. Adjusted Association Between Workforce Well-being Drivers and Four Outcomes among Dentists in HRSA-HCs, 2022-2023

|  | **Dentists (n=722)** | | | | | | | | | | | | | |  |  |  |
| --- | --- | --- | --- | --- | --- | --- | --- | --- | --- | --- | --- | --- | --- | --- | --- | --- | --- |
|  | **Job Satisfaction^4^** | | | | **Engagement^4^** | | | | | **Burnout^4^** | | | | **Intent to Stay^4^** |  |  |  |
|  | **Middle vs Low**  **(T2 vs. T1)** | | **High vs Low**  **(T3 vs. T1)** | | | **Middle vs Low**  **(T2 vs. T1)** | | **High vs Low**  **(T3 vs. T1)** | | **Middle vs Low**  **(T2 vs. T1)** | | **High vs Low**  **(T3 vs. T1)** | | **High vs Low**  **(M2 vs. M1)** |  |  |  |
|  | **RRR**  **(95% CI)*** | | **RRR**  **(95% CI)*** | | | **RRR**  **(95% CI)*** | | **RRR**  **(95% CI)*** | | **RRR**  **(95% CI)*** | | **RRR**  **(95% CI)*** | | **OR**  **(95% CI)*** |  |  |  |
| **Well-being Drivers^4^** |  | |  | | |  | |  | |  | |  | |  |  |  |  |
| Work Team |  | |  | | |  | |  | |  | |  | |  |  |  |  |
| Low (T1) | ref | | | | | | | | | | | | | |  |  |  |
| Middle (T2) |  | |  | | | 2.29 (1.36, 3.85)** | | 1.42 (0.78, 2.59) | | 0.45 (0.25, 0.83)* | | 0.32 (0.16, 0.66)** | | 1.59 (0.93, 2.71) |  |  |  |
| High (T3) |  | |  | | | 2.25 (1.16, 4.38)* | | 2.69 (1.35, 5.35)** | | | | 0.43 (0.22, 0.86)* | | 0.45 (0.19, 1.04) |  |  |  |
| Supervision |  | |  | | |  | |  | |  | |  | |  |  |  |  |
| Low (T1) | ref | | | | | | | | | | | | | |  |  |  |
| Middle (T2) | 1.83 (1.00, 3.37) | | 1.60 (0.71, 3.62) | | |  | |  | | 0.58 (0.31, 1.08) | | 0.31 (0.15, 0.63)** | |  |  |  |  |
| High (T3) | 4.25 (1.68, 10.76)** | | 3.53 (1.21, 10.32)* | | |  | |  | | 0.46 (0.21, 1.02) | | 0.28 (0.11, 0.74)* | |  |  |  |  |
| Leadership |  | |  | | |  | |  | |  | |  | |  |  |  |  |
| Low (T1) | ref | | | | | | | | | | | | | |  |  |  |
| Middle (T2) |  | |  | | | | |  | |  | |  | | 2.55 (1.40, 4.63)** |  |  |  |
| High (T3) |  | |  | | | | |  | |  | |  | | 2.36 (0.88, 6.33) |  |  |  |
| Positive Workplace Culture |  | |  | | |  | |  | |  | |  | |  |  |  |  |
| Low (T1) | ref | | | | | | | | | | | | | |  |  |  |
| Middle (T2) | 2.17 (1.18, 3.99)* | | 2.17 (0.93, 5.07) | | | 2.23 (1.29, 3.84)** | | 1.19 (0.64, 2.22) | |  | |  | | 2.44 (1.31, 4.54)** |  |  |  |
| High (T3) | 2.08 (0.84, 5.19) | | 3.91 (1.32, 11.63)* | | | 1.96 (0.90, 4.27) | | 1.97 (0.86, 4.49) | |  | |  | | 1.28 (0.53, 3.11) |  |  |  |
| Social Support |  | |  | | |  | |  | |  | |  | |  |  |  |  |
| Low (T1) | ref | | | | | | | | | | | | | |  |  |  |
| Middle (T2) |  | |  | | |  | |  | | 1.09 (0.55, 2.15) | | 1.98 (0.84, 4.67) | |  |  |  |  |
| High (T3) |  | |  | | |  | |  | | 1.96 (0.89, 4.29) | | 3.82 (1.44, 10.12)** | |  |  |  |  |
| Recognition |  | |  | | |  | |  | |  | |  | |  |  |  |  |
| Low (T1) | ref | | | | | | | | | | | | | |  |  |  |
| Middle (T2) | 2.37 (1.28, 4.38)** | | 1.34 (0.61, 2.96) | | | 2.03 (1.19, 3.46)** | | 1.11 (0.59, 2.06) | |  | |  | |  |  |  |  |
| High (T3) | 1.98 (0.78, 5.01) | | 1.63 (0.57, 4.62) | | | 1.90 (0.88, 4.12) | | 1.74 (0.78, 3.89) | |  | |  | |  |  |  |  |
| Supportive Health Center Processes |  | |  | | |  | |  | |  | |  | |  |  |  |  |
| Low (T1) | ref | | | | | | | | | | | | | |  |  |  |
| Middle (T2) | 0.67 (0.37, 1.24) | | 1.17 (0.52, 2.62) | | |  | |  | |  | |  | |  |  |  |  |
| High (T3) | 0.31 (0.14, 0.69)** | | 0.45 (0.17, 1.16) | | |  | |  | |  | |  | |  |  |  |  |
| Training Provided |  | |  | | |  | |  | |  | |  | |  |  |  |  |
| Low (T1) | ref | | | | | | | | | | | | | |  |  |  |
| Middle (T2) |  | |  | | |  | |  | |  | |  | |  |  |  |  |
| High (T3) |  | |  | | |  | |  | |  | |  | |  |  |  |  |
| Adequate Resources |  | |  | | |  | |  | |  | |  | |  |  |  |  |
| Low (T1) | ref | | | | | | | | | | | | | |  |  |  |
| Middle (T2) |  | |  | | |  | |  | |  | |  | |  |  |  |  |
| High (T3) |  | |  | | |  | |  | |  | |  | |  |  |  |  |
| Mission Orientation |  | |  | | |  | |  | |  | |  | |  |  |  |  |
| Low (M1) | ref | | | | | | | | | | | | | |  |  |  |
| High (M2) |  | |  | | | 0.91 (0.54, 1.53) | | 1.96 (1.12, 3.45)* | | | |  | |  |  |  |  |
| Meaningfulness |  | |  | | |  | |  | |  | |  | |  |  |  |  |
| Low (M1) | ref | | | | | | | | | | | | | |  |  |  |
| High (M2) | 1.70 (0.92, 3.13) | | 4.46 (2.20, 9.02)*** | | | 1.84 (1.08, 3.16)* | | 3.62 (2.09, 6.26)*** | | 0.60 (0.36, 1.00) | | 0.26 (0.14, 0.50)*** | | 1.89 (1.10, 3.25)* |  |  |  |
| Compensation & Benefits |  | |  | | |  | |  | |  | |  | |  |  |  |  |
| Low (T1) | ref | | | | | | | | | | | | | |  |  |  |
| Middle (T2) | 5.21 (2.90, 9.39)*** | | 7.39 (3.33, 16.41)*** | | |  | |  | |  | |  | | 2.46 (1.46, 4.16)** |  |  |  |
| High (T3) | 2.79 (1.19, 6.55)* | | 11.07 (4.16, 29.40)*** | | | | |  | |  | |  | |  |  |  |  |
| Professional Growth |  | |  | | |  | |  | |  | |  | |  |  |  |  |
| Low (T1) | ref | | | | | | | | | | | | | |  |  |  |
| Middle (T2) | 2.56 (1.41, 4.63)** | | 3.10 (1.42, 6.77)** | | |  | |  | | 0.76 (0.43, 1.35) | | 0.42 (0.21, 0.83)* | | 1.93 (1.10, 3.40)* |  |  |  |
| High (T3) | 0.95 (0.41, 2.18) | | 3.73 (1.51, 9.25)** | | |  | |  | | 0.47 (0.24, 0.90)* | | 0.21 (0.09, 0.49)*** | | 3.41 (1.48, 7.83)** |  |  |  |
| Workload |  | |  | | |  | |  | |  | |  | |  |  |  |  |
| Low (T1) | ref | | | | | | | | | | | | | |  |  |  |
| Middle (T2) | 0.71 (0.31, 1.58) | | 0.43 (0.18, 1.05) | | |  | |  | | 2.37 (1.38, 4.06)** | | 3.21 (1.48, 6.93)** | | |  |  |  |
| High (T3) | 0.29 (0.12, 0.67)** | | 0.14 (0.05, 0.40)*** | | |  | |  | | 3.12 (1.44, 6.76)** | | 11.62 (4.60, 29.36)*** | | |  |  |  |
| Work-life Balance |  | |  | | |  | |  | |  | |  | |  |  |  |  |
| Low (T1) | ref | | | | | | | | | | | | | |  |  |  |
| Middle (T2) | 2.16 (1.17, 3.96)* | | 2.08 (0.95, 4.55) | | |  | |  | | 0.56 (0.31, 1.00) | | 0.17 (0.09, 0.35)*** | |  |  |  |  |
| High (T3) | 1.44 (0.68, 3.08) | | 4.97 (2.10, 11.78)*** | | |  | |  | | 0.29 (0.15, 0.54)*** | | 0.10 (0.04, 0.21)*** | |  |  |  |  |
| Moral Distress |  | |  | | |  | |  | |  | |  | |  |  |  |  |
| Low (T1) | ref | | | | | | | | | | | | | |  |  |  |
| Middle (T2) | 0.49 (0.23, 1.03) | | 0.39 (0.17, 0.90)* | | | 0.52 (0.30, 0.92)* | | 0.36 (0.20, 0.66)** | | 1.64 (0.96, 2.79) | | 2.19 (1.05, 4.55)* | |  |  |  |  |
| High (T3) | 0.31 (0.14, 0.72)** | | 0.21 (0.07, 0.58)** | | | 0.36 (0.19, 0.70)** | | 0.22 (0.11, 0.44)*** | | 2.86 (1.27, 6.46)* | | 5.95 (2.31, 15.32)*** | | |  |  |  |
| **Variables** |  | |  | | |  | |  | |  | |  | |  |  |  |  |
| Age |  | |  | | |  | |  | |  | |  | |  |  |  |  |
| Under 40 | 0.44 (0.25, 0.77)** | | 0.42 (0.21, 0.84)* | | | 0.54 (0.34, 0.88)* | | 0.40 (0.24, 0.67)** | | 1.92 (1.16, 3.16)* | | 4.41 (2.36, 8.21)*** | | 0.66 (0.41, 1.07) |  |  |  |
| 40 and older | ref | | | | | | | | | | | | | |  |  |  |
| Gender |  | |  | | |  | |  | |  | |  | |  |  |  |  |
| Male |  | |  | | | 0.93 (0.57, 1.50) | | 0.55 (0.32, 0.94)* | |  | |  | |  |  |  |  |
| Female/Other^1^ | ref | | | | | | | | | | | | | |  |  |  |
| Race/Ethnicity |  | |  | | |  | |  | |  | |  | |  |  |  |  |
| White Non-Hispanic | ref | | | | | | | | | | | | | |  |  |  |
| All Other Race^2^ Ethnicities | 1.21 (0.67, 2.17) | | 1.11 (0.55, 2.25) | | | 1.17 (0.73, 1.90) | | 2.01 (1.19, 3.39)** | | 0.52 (0.32, 0.83)** | | 0.34 (0.19, 0.61)*** | | 1.11 (0.68, 1.80) |  |  |  |
| Marital Status |  | |  | | |  | |  | |  | |  | |  |  |  |  |
| Married | 0.97 (0.45, 2.05) | | 1.35 (0.54, 3.38) | | | 1.01 (0.55, 1.86) | | 1.20 (0.62, 2.29) | | 1.09 (0.55, 2.17) | | 1.39 (0.62, 3.11) | | 0.83 (0.45, 1.52) |  |  |  |
| Never Married | ref | | | | | | | | | | | | | |  |  |  |
| Previously Married/Separated | 3.53 (1.18, 10.57)* | | 0.95 (0.21, 4.30) | | | 1.31 (0.49, 3.49) | | 1.04 (0.36, 2.97) | | 0.57 (0.21, 1.53) | | 0.24 (0.06, 0.88)* | | 0.94 (0.35, 2.54) |  |  |  |
| Urban/Rural |  | |  | | |  | |  | |  | |  | |  |  |  |  |
| Rural | 1.38 (0.74, 2.57) | 0.51 (0.24, 1.09) | |  | | |  | |  | |  | | 0.56 (0.32, 0.97)* | |  |  |  |
| Urban | ref | | | | | | | | | | | | | |  |  |  |
| Health Center Size |  | |  | | |  | |  | |  | |  | |  |  |  |  |
| Small |  | |  | | | 0.46 (0.23, 0.96)* | | 0.45 (0.20, 0.99)* | |  | |  | | 0.24 (0.11, 0.51)*** |  |  |  |
| Large | ref | | | | | | | | | | | | | |  |  |  |
| Health Center Funding |  | |  | | |  | |  | |  | |  | |  |  |  |  |
| CHC only or Look Alike^3^ |  | |  | | | 1.83 (1.15, 2.90)* | | 1.68 (1.02, 2.78)* | |  | |  | | 1.78 (1.08, 2.93)* |  |  |  |
| Multiply funded | ref | | | | | | | | | | | | | |  |  |  |
| HRSA Region |  | |  | | |  | |  | |  | |  | |  |  |  |  |
| Region 1 (CT, ME, MA, NH, RI, VT) |  | |  | | |  | |  | | 0.47 (0.22, 0.98)* | | 0.37 (0.15, 0.92)* | | 0.41 (0.20, 0.83)* |  |  |  |
| Region 2 (NJ, NY, PR, Virgin Islands) |  | | 0.31 (0.11, 0.90)* | | |  | |  | | | |  | |  |  |  |  |
| Region 3 (DE, DC, MD, PA, VA, WV) |  | |  | | |  | |  | |  | |  | |  |  |  |  |
| Region 4 (AL, FL, GA, KY, MS, NC, SC, TN) |  | |  | | |  | |  | | 0.36 (0.16, 0.79)* | | 0.29 (0.11, 0.76)* | | 0.46 (0.22, 0.97)* |  |  |  |
| Region 5 (IL, IN, MI, MN, OH, WI) |  | |  | | |  | |  | |  | |  | |  |  |  |  |
| Region 6 (AR, LA, NM, OK, TX) |  | |  | | |  | |  | |  | |  | |  |  |  |  |
| Region 7 (IA, MO, NE, KS) | 2.23 (0.69, 7.21) | | 5.64 (1.38, 23.12)* | | | 0.32 (0.13, 0.79)* | | 0.23 (0.07, 0.71)* | |  | |  | |  |  |  |  |
| Region 8 (CO, MT, ND, SD, UT, WY) |  | |  | | |  | |  | |  | |  | |  |  |  |  |
| Region 9 (AZ, CA, HI, NV, American Samoa, Guam, Northern Mariana Islands, Federated States of Micronesia, Marshall Islands, Palau) | ref | | | | | | | | | | | | | |  |  |  |
| Region 10 (AK, ID, OR, WA) |  | |  | | |  | |  | |  | |  | |  |  |  |  |

Source: Health Resources and Services Administration (HRSA) Health Center Workforce Well-being Survey, 2022-2023.

Note: ^1^ Female/Other, female, female-to-male (FTM)/transgender male/trans man, male-to=female (MTF)/transgender female/trans women, genderqueer, neither exclusively male or female, something else, and don’t know/not sure based on gender identity.

^2^ All Other Race Ethnicities, Hispanic, Black Non-Hispanic, and Other Non-Hispanic.

^3^ CHC only or Look Alike, Community Health Center (CHC) Only.

^4^ Survey items were assessed on a 6-point Likert scale ranging from (1) strongly disagree to (6) strongly agree and reverse scored as needed so that higher composite scores represented greater levels of the construct named. Higher burnout, workload, and moral distress scores indicate less favorable well-being outcomes or workplace conditions. T1, T2, and T3 denote the low, middle, and high tertiles, respectively; M1 and M2 denote the low and high dichotomized categories, respectively.
